# Sequence variation, common tissue expression patterns and learning models: a genome-wide survey of vertebrate ribosomal proteins

**DOI:** 10.1093/nargab/lqaa088

**Published:** 2020-11-06

**Authors:** Konstantinos A Kyritsis, Christos A Ouzounis, Lefteris Angelis, Ioannis S Vizirianakis

**Affiliations:** Laboratory of Pharmacology, School of Pharmacy, Aristotle University of Thessaloniki, GR-54124 Thessalonica, Greece; Biological Computation & Process Laboratory, Chemical Process & Energy Resources Institute, Centre for Research & Technology Hellas, GR-57001 Thessalonica, Greece; Biological Computation & Process Laboratory, Chemical Process & Energy Resources Institute, Centre for Research & Technology Hellas, GR-57001 Thessalonica, Greece; Department of Informatics, Aristotle University of Thessaloniki, GR-54124 Thessalonica, Greece; Department of Informatics, Aristotle University of Thessaloniki, GR-54124 Thessalonica, Greece; Laboratory of Pharmacology, School of Pharmacy, Aristotle University of Thessaloniki, GR-54124 Thessalonica, Greece; FunPATH (Functional Proteomics and Systems Biology Research Group at AUTH) Research Group, KEDEK—Aristotle University of Thessaloniki, Balkan Center, GR-57001 Thessalonica, Greece; Department of Life and Health Sciences, University of Nicosia, CY-1700 Nicosia, Cyprus

## Abstract

Ribosomal genes produce the constituents of the ribosome, one of the most conserved subcellular structures of all cells, from bacteria to eukaryotes, including animals. There are notions that some protein-coding ribosomal genes vary in their roles across species, particularly vertebrates, through the involvement of some in a number of genetic diseases. Based on extensive sequence comparisons and systematic curation, we establish a reference set for ribosomal proteins (RPs) in eleven vertebrate species and quantify their sequence conservation levels. Moreover, we correlate their coordinated gene expression patterns within up to 33 tissues and assess the exceptional role of paralogs in tissue specificity. Importantly, our analysis supported by the development and use of machine learning models strongly proposes that the variation in the observed tissue-specific gene expression of RPs is rather species-related and not due to tissue-based evolutionary processes. The data obtained suggest that RPs exhibit a complex relationship between their structure and function that broadly maintains a consistent expression landscape across tissues, while most of the variation arises from species idiosyncrasies. The latter may be due to evolutionary change and adaptation, rather than functional constraints at the tissue level throughout the vertebrate lineage.

## INTRODUCTION

Ribosomes constitute indispensable molecular machines composed of two distinct small (SSU) and large (LSU) ribonucleoprotein subunits, that catalyze protein synthesis in prokaryotes and eukaryotes alike. In eukaryotes, cytoplasmic ribosome biogenesis constitutes a highly complicated, costly and well-coordinated process, which requires the participation of four ribosomal RNAs (rRNAs), 80 core ribosomal proteins (RPs) (79 in yeast) and hundreds of ribosome-associated factors (proteins and non-coding RNAs) that mediate SSU and LSU translocation, maturation and quality control (1–4). Studies of ribosome structures have revealed a distinct mode of evolution, with archaeal and eukaryotic ribosomes sharing more similarities than their bacterial counterparts (5,6). The conserved ribosome core consists of 33 universally conserved RPs and rRNA with a length of ∼4400 bases and regions critical for translation (7–11). For eukaryotes in particular, the SSU holds the ribosomal decoding center (DC) for messenger RNA (mRNA), performs mRNA scanning during translation initiation and comprises 18S rRNA and 33 RPs, while the LSU contains the peptidyl-transferase (PTC) and the GTPase-centers, and comprises three rRNA molecules (28S, 5.8S and 5S) and 47 RPs. Ribosome is a dynamic ribonucleoprotein complex that transitions through different conformations and functional states during the four phases (initiation, elongation, termination and recycling) of the translation process (12–14).

Compared to the bacterial form, eukaryotic ribosomes are larger and more complex with respect to both rRNA and protein content. Specifically, thousands of additional nucleotides (∼2650 in human) form rRNA expansion segments, while 26 additional RPs as well as amino acids (∼2452 in human) contribute to the formation of eukaryotic-specific RP moieties (5,8). Eukaryote-specific rRNA, RP expansion segments and additional RPs shape a flexible layer, which is primarily located on the ribosome surface. This layer facilitates interactions with various eukaryotic translation factors and is part of a network of translational regulation mechanisms that are under intense investigation (5,15–16). Importantly, structural differences are not limited to comparisons between bacterial and archaeal/eukaryotic ribosomes. Ribosome structural studies in various eukaryotic models have revealed the presence of additional intertwined RNA–RNA and RNA–protein layers in human ribosomes, in contrast to their counterparts in *Saccharomyces cerevisiae* (17), *Tetrahymena thermophila* (18,19) and *Drosophila melanogaster* (5), supporting the notion of continuous evolutionary adaptation even within the same domain of life.

Interestingly, the roles of RPs extend well beyond mere structural constituents of the ribosome. This has been demonstrated through heterozygous mutations affecting the expression of specific RPs (haploinsufficiency) leading to congenital diseases both in mouse and human. Prominent examples are *RPS14/uS11*, *RPSA/uS2* and *RPL38/eL38* genes with mutations associated with 5q- myelodysplastic syndrome (20), isolated congenital asplenia (21) and skeletal defects during embryogenesis, including perturbations in the formation of the axial skeleton (22). Perhaps the most well-known case of ribosomopathies is Diamond-Blackfan anemia (DBA), a genetic disease with mutations in 19 different RPs being responsible for more than 50% of the cases (23). Although DBA patients display various pathological phenotypes, depending on the RP gene carrying the mutation and their genetic background (24–26), they share tissue-specific symptoms like anemia, due to inhibition of erythropoiesis and defects in skeletal development (25,27) as well as pre-disposition to the development of specific types of cancer (28,29). Furthermore, a subset of RPs has been shown to interact and block the activity of Mdm2 (Hdm2 in humans), a E3 ubiquitin-protein ligase responsible for the ubiquitination and proteolysis of p53 anti-tumor protein. During nucleolar stress, ribosome-free RPs induce inhibition of cell cycle progression and apoptosis through increased levels of p53—reviewed in (30,31), with the RPL5/uL18, RPL11/uL5 and 5S rRNA complex playing a pivotal role in this checkpoint of cellular growth and proliferation (32–35). Abnormal activation of p53 due to RP haploinsufficiency has been associated with the pathogenesis of several ribosomopathies (36,37).

Discoveries of specialized roles for RPs were accompanied by a series of studies exploring the RP expression patterns by means of reverse transcription quantitative PCR (RT-qPCR), DNA microarray and RNA-sequencing (RNA-seq) analysis of human and mouse tissues. It was thus established that a subgroup of paralog RPs exhibit highly tissue-specific expression patterns, questioning the conservation of ribosome structure and function. Specifically, RPL3L (38–41) and the set of RPL10L (40,42–43)/RPL39L (39,44–45)/RPS4Y2 (46) have been found to be almost exclusively expressed in skeletal/cardiac muscle and testis, respectively. Functional implications of RP paralog tissue enrichment have emerged: RPL3L was downregulated in striated muscle in response to hypertrophic stimuli, contrary to its paralog RPL3/uL3 and acted as an inhibitor of muscle growth *in vitro* (41). Similarly, RPL10L is indispensable for spermatogenesis, compensating for the loss of its paralog, RPL10/uL16, due to meiotic sex chromosome inactivation. Lack of RPL10L impaired ribosome biogenesis and cell cycle progression in spermatocytes and resulted in male infertility *in vivo* (43).

Apart from tissue-enriched RP paralogs, conflicting results have been reported regarding the expression of most RPs. It has been reported that tissue-specific expression patterns exist for subgroups of RPs across different mammalian tissues (47–50). Yet, other studies conclude that the expression of RPs, excluding RP paralogs, is highly consistent, positively correlated and differs solely across samples of different tissue origin (39,40). In fact, analysis of microarray data across 22 murine tissues established RPs as housekeeping genes, considering their universal presence in all tissues, maintaining the important distinction that they should not be considered as reference genes for quantification assays, as they present tissue-dependent expression differences (39).

Given the importance of ribosomal structure and function in cell physiology, we endeavour to systematically explore the levels of sequence conservation of RPs in vertebrate species for which there is abundance of gene expression information across different tissues and contrast those. The goal of this comprehensive survey is to quantify variation of RPs in terms of sequence-structure and function and assess the extent at which tissue specificity manifests itself in particular instances for ribosomal genes, across vertebrates.

To this end, we collected, compiled and curated RP sequences in eleven vertebrates and up to 33 tissues. Exhaustive sequence comparisons reveal a previously unknown, somewhat unexpected variation among vertebrate RPs. Furthermore, analysis of gene expression indicates that most RPs appear to share a common pattern of gene expression that is conserved and fluctuates similarly for the same tissue across different species, with several important exceptions involving paralog RP genes.

## MATERIALS AND METHODS

### Harvesting and curation of vertebrate RP sequences

RP gene symbols of both the previous and the new nomenclature (9) were used to perform manual queries to the NCBI RefSeq Database in order to retrieve the respective RP reference sequences for each of the eleven vertebrate species, namely *Homo sapiens* (human), *Pan troglodytes* (chimpanzee), *Pongo abelii* (Sumatran orangutan), *Macaca mulatta* (rhesus macaque), *Mus musculus* (house mouse), *Monodelphis domestica* (gray short-tailed opossum), *Ornithorhynchus anatinus* (platypus), *Gallus gallus* (chicken), *Anolis carolinensis* (arboreal anole lizard), *Xenopus tropicalis* (western clawed frog) and *Danio rerio* (zebrafish) (51). For one or more vertebrate species, 23 RPs failed to return ortholog sequences by either annotation queries or alignments using BLASTP and the respective 90 RP *H. sapiens* protein sequence, as queries (52). For these missing RPs, *H. sapiens* orthologs were used again to perform queries using TBLASTN to identify DNA sequences with statistically significant similarity (*e*-value < 0.05), subsequently translated and added to complete our RP protein sequence collection. The RP collection was manually curated and annotated by setting unique IDs specifying gene name, NCBI RefSeq ID and vertebrate species. The RP protein sequence of the largest size was marked by the term ‘ref_’ (as reference) and in case of additional sequences, they were marked and enumerated using the term ‘iso_’ (isoforms). In total, 1083 RP sequences were assimilated into our sequence data collection. Confirming the validity of our approach, we noted that several *O. anatinus* translated RPs we had marked, were also incorporated into a more recent release (NCBI *Ornithorhynchus anatinus* Annotation Release 104). Heatmaps for phylogenetic profiling (also expression, see below) were created with ComplexHeatmap (53).

Total proteomes for the eleven vertebrate species were retrieved from Ensembl (release 100) (54). The CAST-masked RP collection was used as query for sequence searches against vertebrate proteomes, performed using BLASTP (*e*-value < 0.05) (52). A total of 210 RP protein sequences do not return BLASTP hits for at least one vertebrate proteome. Overall, 19 RPs are missing BLASTP hits from at least one vertebrate proteome (see ‘Results’ section).

### Sequence comparison and clustering

Automatic all-versus-all sequence comparison was performed for the entire RP protein sequence collection. Low-complexity masking was applied to the RP sequences using CAST (55) and comparisons were performed using BLASTP (*e*-value < 0.05) (52). Pairwise lists of significant hits, with a minimum threshold of 50% for identity score, were used to form sequence similarity networks and Markov-chain clustering (MCL) (inflation = 1.8, minimum three nodes per cluster) (56) was applied using Biolayout 3D Express (57). This *e*-value threshold is justified by the fact that the searches are not blind but supervised, i.e. the query set is known (RPs) and we go for high sensitivity (no false negatives), and ‘low precision’ (i.e. potentially some false positives, for which we have none). Therefore, it is not problematic to run searches when the query as well as the target sets are well-defined, as in this case.

### Multiple sequence alignment of RPL29/eL29, RPL14/eL14 and RPL4/uL4 orthologs

Multiple sequence alignment (MSA) for RPL29/eL29, RPL14/eL14 and RPL4/uL4 using ortholog sequences from our RP collection was performed with MUSCLE (58) and MSA (59). Furthermore, additional MSA for RPL29/eL29, RPL14/eL14 and RPL4/uL4 using ortholog sequences retrieved from NCBI Eukaryotic Genome Annotation Pipeline (60) was performed with COBALT (61) and visualized with Jalview (62) to corroborate our analysis with a more extensive range of taxa (not shown). Conserved domains within the aligned regions were identified using Conserved Domain Database (63). For human RP orthologs, these are regions 1–53 for RPL29/eL29, 1–131 for RPL14/eL14 and 1–341 for RPL4/uL4, defined by the local MSA of our RP collection.

### Ribosome structure visualization

A discrete color coding scheme, based on the highest identity score of each RP cluster, was applied to the respective RP chains of entry 4V6X from PDB, a high-resolution cryo-EM structure of *H. sapiens* 80S ribosomes in complex with the translation factor eEF2, E-site transfer RNA and Stm1-like proteins, and was created based on high-resolution cryo-electron-microscopy density maps (5). Ribosome visualization and rendering were executed using UCSF Chimera (64).

### Expression profiles in GTEx dataset

RP expression values for non-disease human tissues were retrieved from the Genotype-Tissue Expression project (GTEx, version 8). GTEx contains gene expression data (RNA-seq) from 17 382 samples (extracted from 54 tissues sites of 948 donors) (65). Expression values (Transcript Per Million; TPM) were scaled between 0 and 1 using Min–Max normalization for each RP. Dimensionality reduction of scaled RP expression data, following removal of gender-associated RPL10L, RPS4X/eS4, RPS4Y1/2, RPL39L and RPL26L1 to avoid bias, was performed using t-Distributed Stochastic Neighbor Embedding (t-SNE) (66). Two- and three-dimensional visualizations for t-SNE results were performed with plotly (67). GTEx samples were assigned to 33 tissue categories.

### Tissue classification learning models based on GTEx

RP expression values from GTEx (TPM) were used to train multi-classification learning models to predict the type of tissue assigned to each sample. During pre-processing, gender-associated paralogs RPL10L, RPS4X/eS4, RPS4Y1/2, RPL39L and RPL26L1 were removed. Four learning models, that include Logistic Regression (one-versus-rest scheme for multiclassification), Support-vector machine with Linear (LinearSVC) or Gaussian (SVC) kernel and Random Forest, were trained using scaled RP expression values. All multi-classification learning models in the present study were created using scikit-learn (68). To evaluate the ability of learning models for accurate prediction of tissue categories, a nested cross-validation strategy was adopted: the expression dataset was split three times into training (90%) and testing (10%) datasets (outer loop). For each split, learning models were trained based on the training dataset, following Min-Max scaling for each RP record, using 5-fold cross-validation and optimizing the model's parameters with grid-searching (inner loop). For the final evaluation of model performance, we calculated the arithmetic mean and standard deviation of accuracy, F1-score and Matthew's correlation coefficient (MCC) measurements for all three splits.

### Analysis of expression profiles in vertebrates

For the analysis of RP expression patterns across vertebrate species and tissues, we utilized RNA-Seq data of 68 RPs (corrected Reads Per Kilobase Million; cRPKM) (69) in two combinations to maximize coverage, respectively: i) six common tissues for five species, i.e. maximum number of common tissues, and ii) three common tissues for seven species, i.e. maximum number of common species. Expression values were scaled between 0 and 1 using Min–Max for i) each organism and ii) each RP, before being reordered per tissue for comparisons. Note that the choice of the eleven vertebrate species was partly imposed by the availability of genome-wide tissue expression data, generated for splicing variants (69).

In order to evaluate the ability of learning models (Logistic Regression, LinearSVC, SVC and Random Forest) trained on *H. sapiens* RP expression profiles to predict the tissue type of different vertebrate species, we utilized the total RP expression values from GTEx (TPM) as training dataset. For each species, RP expression values of different tissues (cRPKM) were used as testing dataset, to evaluate model performance on that species. Due to missing RP expression values, only a subset of the training set was used for each species matching RPs and tissues present in its test dataset. Prior to the analysis, both training and test datasets were scaled between 0 and 1 using Min–Max normalization. Models were trained using 5-fold cross-validation and parameters were optimized with grid-searching. Model performance was evaluated using accuracy, F1-score and MCC measurements, as above.

## RESULTS

### RPs for 11 vertebrate species are clustered in 78 homologous families

**Figure 1.**
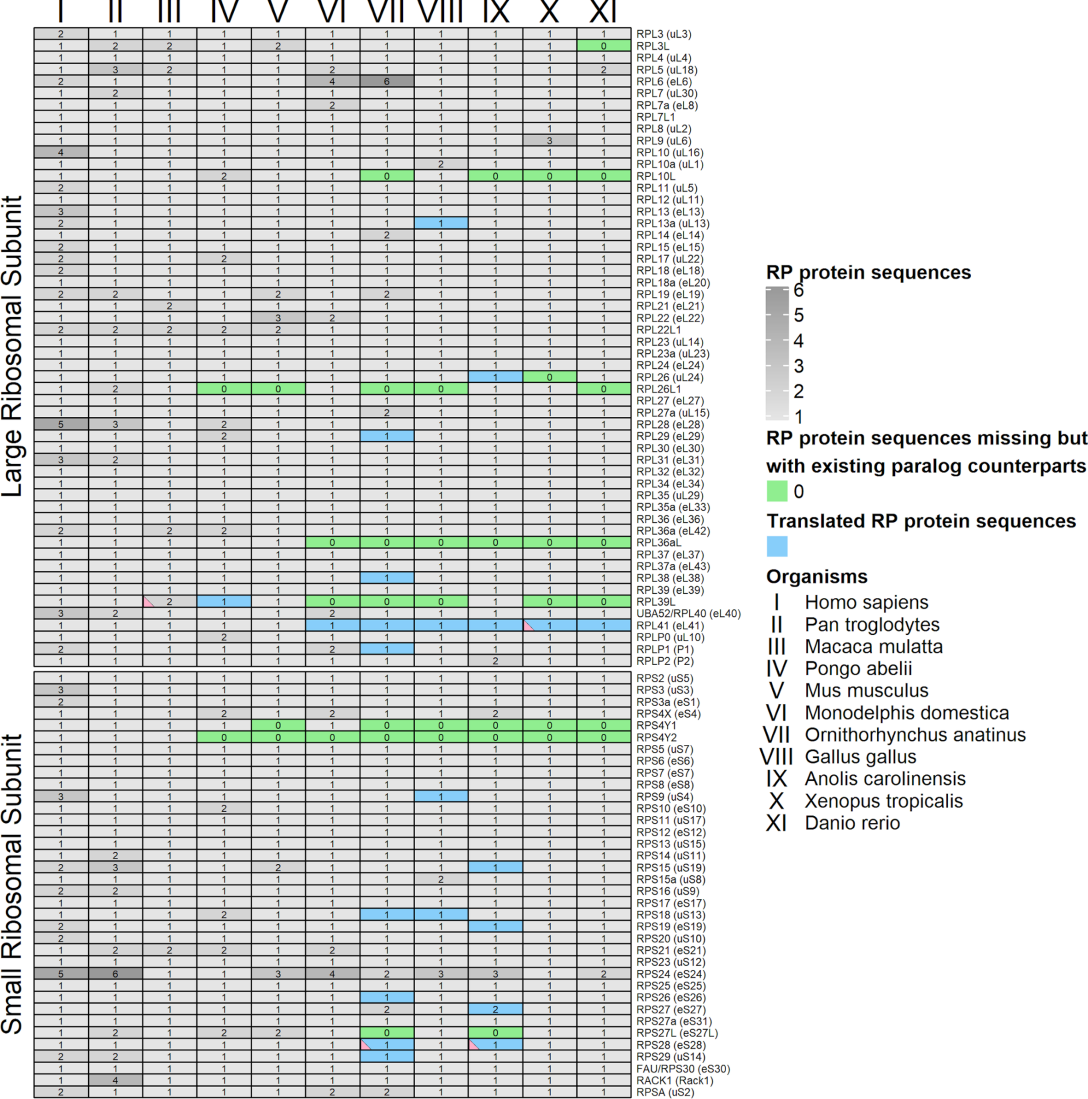
Detailed description of protein sequence collection for 90 RPs of 11 representative vertebrate species. The number of RefSeq protein isoforms, for each RP in each species, is displayed with a gray color scale. RP paralog pairs for which only one paralog was detected are shown in green. RPs that were retrieved from translated nucleic acid sequences are shown in blue. Four RP sequences that failed to be assigned to one of the 78 RP family clusters with MCL, are marked with pink triangle.

To obtain a well-defined collection of vertebrate RP protein sequences, we systematically searched, manually collected and curated 1083 RP sequences for eleven representative vertebrate species, which cover a wide range of vertebrate evolution. The final collection of RPs and their variants has been encoded using a bespoke system (see ‘Materials and Methods’ section, 90 reference human RPs) and made available (Figure 1 and Table 1). Several of the missing RPs are attributed to paralog RP genes, like RPS4Y2, a paralog of RPS4X/eS4 which is found solely in primates (70). Nevertheless, the absence of the highly conserved RPs has prompt us to explore their presence in the respective Ensembl vertebrate proteomes (release 100) (54), by means of local protein alignment using our manually curated RP collection. We detected and report RP protein sequences missing from Ensembl vertebrate proteomes. We discovered 19 RPs absent in at least one of the vertebrate proteomes used here (Supplementary Table S1). As RPs are highly conserved, and indispensable for ribosome biogenesis and translation, we managed to identify a number of missing protein sequences from the complement of a species, included for completeness. Overall, we present a comprehensive collection of RP protein sequences for eleven vertebrates that forms the basis for all subsequent analyses reported herein.

**Table 1.** Nomenclature of the 11 representative vertebrate species, selected for harvesting and curation of their RP sequences

Species name	Number of identified RPs	Number of RP protein sequence (isoforms)
*A. carolinensis (lizard)*	87	90
*D. rerio (zebrafish)*	83	85
*G. gallus (chicken)*	85	89
*H. sapiens (human)*	90	129
*M. mulatta (macaque)*	90	97
*M. domestica (opossum)*	87	101
*M. musculus (mouse)*	87	96
*O. anatinus (platypus)*	84	94
*P. troglodytes (chimpanzee)*	90	116
*P. abelii (orangutan)*	89	100
*X. tropicalis (frog)*	84	86

The number of identified ortholog RPs as well as the total number of RP sequences, including different protein isoforms of the same RP, per species are also shown here.

Subsequently, we performed cross comparisons using our RP protein sequence collection, in an all-versus-all scheme, after first masking low-complexity regions for all RPs. Sequence comparison results were organized into a pairs list of significant hits, with a minimum threshold of 50% identity score being applied to avoid spurious hits between non-ortholog RPs. Through the application of MCL (56), RPs were assigned to 78 distinct protein family clusters (see next section). As expected, there is a clear separation of ortholog RPs, which were assigned to distinct clusters either i) orthologs only or ii) with their respective paralogs. Notably, four RP protein sequences failed to be assigned into their respective ortholog clusters. For three of those sequences, RPS28-like_Anolis_ref_0 (NW_003338769.1; Translated genomic DNA), RPS8_Ornithorhynchus_ref_0 (XP_016081540.1; low quality protein, obsolete entry, last accessed 19 June 2020) and RPL41-like_Xenopus_ref_0 (NC_030685.1; Translated genomic DNA), exclusion from clusters can be attributed to low quality of protein prediction and short length which in combination with low complexity masking results in low identity scores. Finally, the fourth instance, protein sequence RPL39L_Macaca_ref_0 (XP_014987924.1), originally included due to its genome annotation, presents no evidence that it is an ortholog of RPL39L or RPL39/eL39 and should be considered an erroneous annotation record.

### Vertebrate RPs are >80% identical between zebrafish and human, with six exceptions

**Figure 2.**
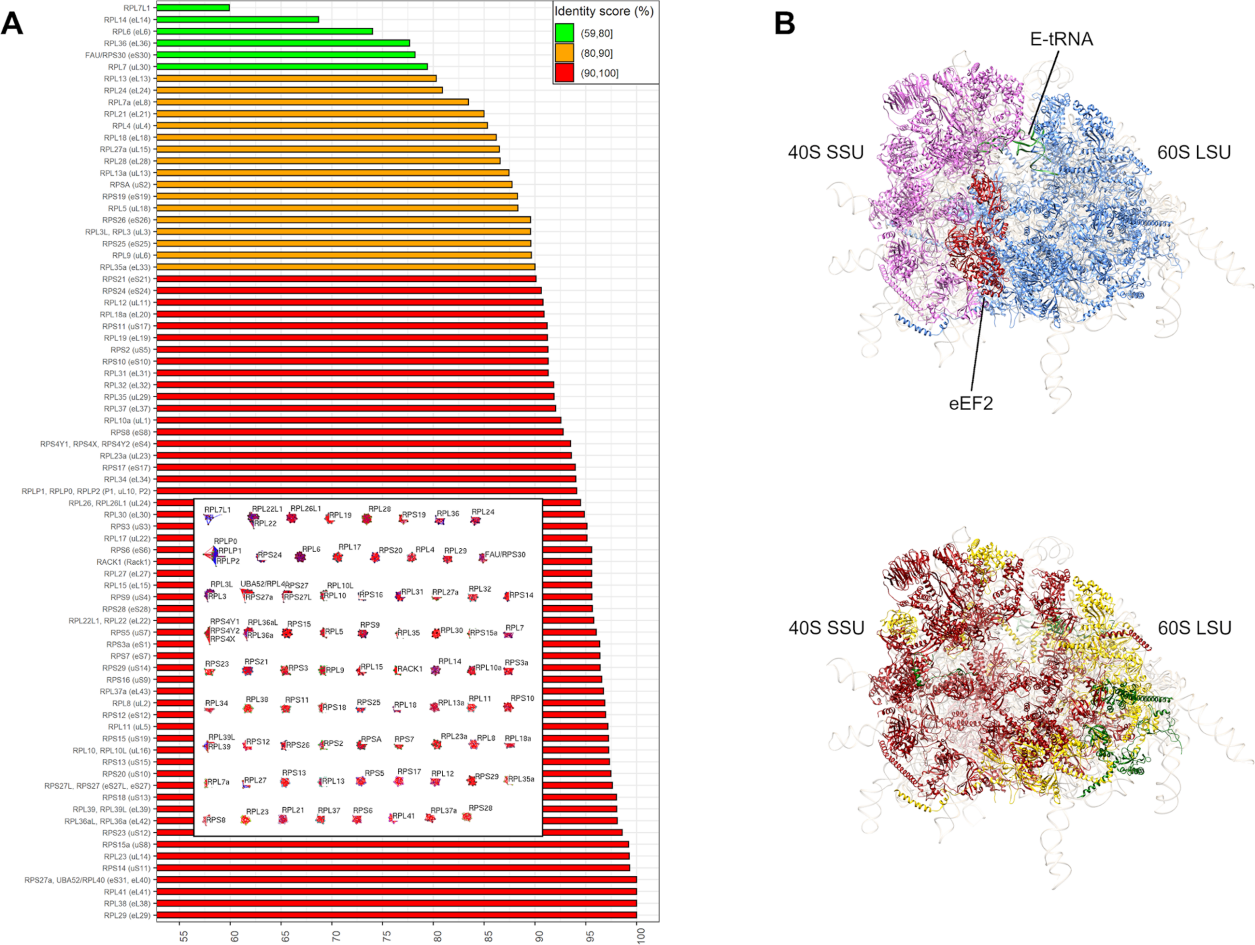
Within-cluster identity score values (%) (*x*-axis) between the *Homo sapiens* and *Danio rerio* ortholog RPs, for each RP cluster (see ‘Materials and Methods’ section). (**A**) Bar plot of increasing within-cluster identity scores (*x*-axis) for each cluster (*y*-axis). Network of vertebrate RP sequence clusters is shown in the centre. Sequence similarity networks were formed and MCL was applied using Biolayout 3D Express (57). (**B**) 3D structure of *H. sapiens* ribosome based on 4V6X model from PDB database (5): (up) SSU and LSU RPs are highlighted with magenta and blue, respectively, while eEF2 factor (red) and E-tRNA structures (green) are also shown; (down) SSU and LSU RPs colored based on within-cluster identity scores. RPs are colored based on a within-cluster identity score scale with green for 59–80%, orange for 80–90% and red for 90–100%. rRNAs are shown as light gray and transparent. Image was created using UCSF Chimera (64).

To provide an accurate estimation of protein sequence conservation across vertebrate species and map it to the three-dimensional structure of the ribosome, *H. sapiens* (human) and *D. rerio* (zebrafish) RP protein sequences from each cluster were selected and compared (Figure 2). Human and zebrafish represent the most distant vertebrate species in our set and both have a record of curated protein sequences (51,71). For each cluster, the pair of human and zebrafish RPs with the highest identity score was selected and was used as metric of estimation of sequence conservation within the vertebrate lineage. We observed distinct differences between the estimated RP sequence conservation levels, range from ∼60 to 100% identity score (Figure 2A). As expected, most RPs exhibit high sequence identity scores (>80%), except for RPL7L1, RPL14/eL14, RPL6/eL6, RPL36/eL36, FAU/RPS30-precursor/eS30 and RPL7/uL30. Also, comparison of within-cluster identity score ranks between SSU and LSU RPs, after removing the cluster of RPS27a/eS31 and UBA52/RPL40-precursor/eL40, that contains RPs from both ribosomal subunits, shows a small (∼4%) but significant increase in identity score for SSU RPs (*P*-value < 0.05; non-parametric, two-sided Wilcoxon test). This observation could be related to the slightly higher proportion of universal RPs in SSU both in prokaryotes and eukaryotes, due to the higher conservation of the SSU rRNA (7) (Figure 2B).

Our analysis illustrates evolutionary relations between RPs and implies that RP-dependent translation regulation strategies may be associated with extreme levels of sequence conservation. Examples of taxon-specific expansion in vertebrates are provided with more details in Supplementary Data (and Supplementary Figures S1–4). Our findings are also corroborated by experimental evidence as outlined above, for particular cases at the extremes of sequence conservation.

### Sequence variability does not affect a correlated mode of expression for most human RP genes

To investigate possible tissue-specific expression patterns of RPs, we utilized RNA-seq data from the GTEx database v8 (65). Inspection of RP expression profiles in GTEx non-disease human tissues confirmed the known tissue-enrichments (Supplementary Figure S5) displayed by RPL3L (38–41) in skeletal and cardiac muscle as well as RPL10L (40,42–43), RPL39L (39,44–45) and RPS4Y2 (46) in testis (not shown). Interestingly, RPL26L1, a RP paralog of RPL26/uL24, was also found enriched in testis but the functional significance of its overexpression has yet to be described. For RPL7L1 and RPS27L, despite lack of information regarding tissue enrichment, there is over-expression in samples of cultured fibroblasts comparted to RPL7/uL30 and RPS27/eS27, respectively—of unknown significance.

**Figure 3.**
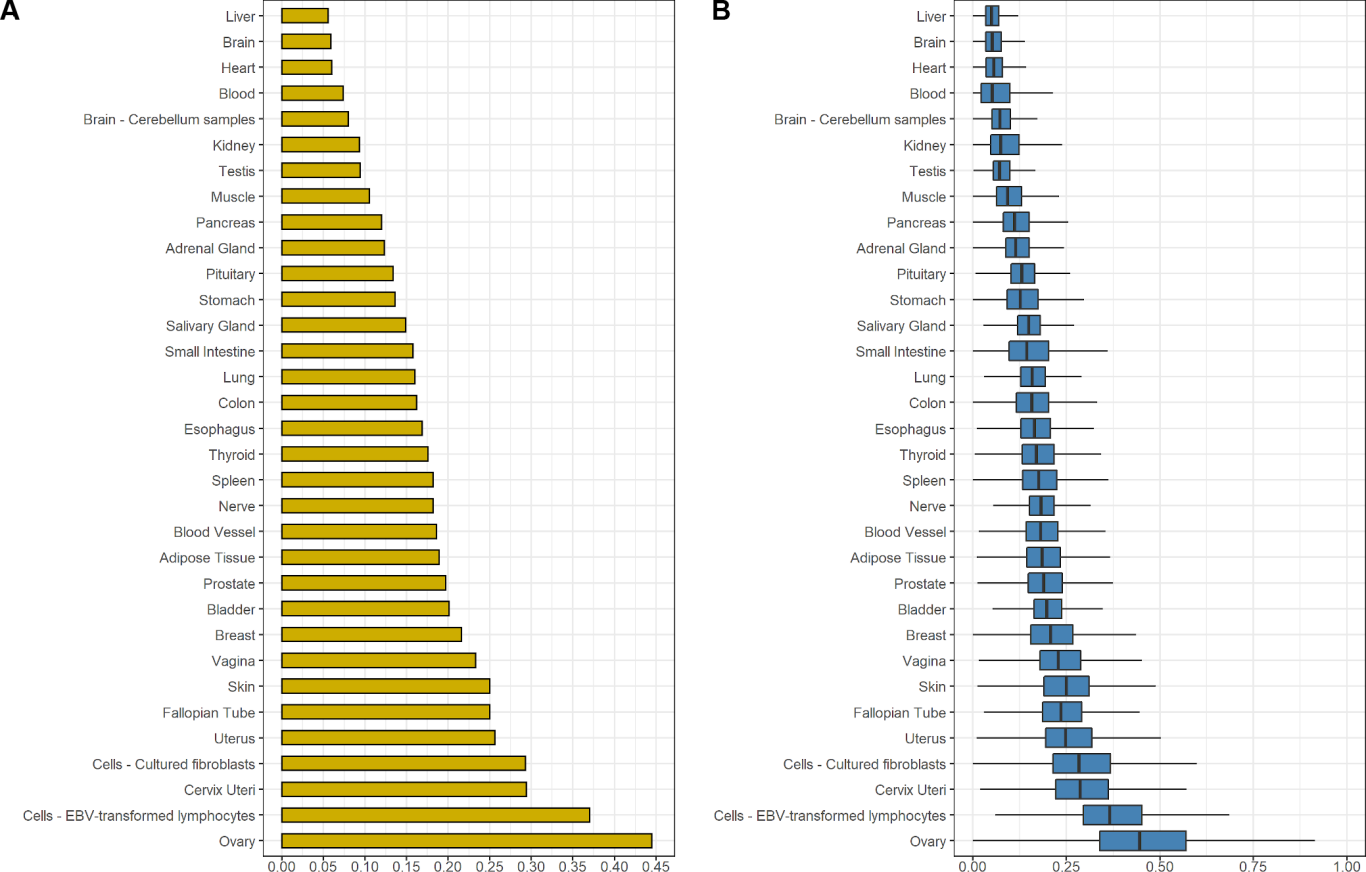
RP expression levels in 33 human tissue categories of GTEx project (65). (**A**) Bar plot of arithmetic mean for the expression values of all RPs (yellow) for each tissue category. (**B**) Boxplot of expression values of all RPs for each tissue category (blue). Expression values (TPM) were scaled between 0 and 1 using Min–Max normalization for each RP. Outliers were removed from boxplots.

Dimension reduction (t-SNE) of samples based on RP expression, separated them into distinct clusters according to their tissue (Supplementary Figures S6 and 7), as was previously reported (72). This led us to assign samples into 33 different categories, notably separating samples of similar tissue origin (Supplementary Figures S8–10). This includes cerebellum samples, which display higher average RP expression than the rest of the samples with brain origin, and tumorigenic *in vitro* cell cultures (EBV-transformed lymphocytes and cultured fibroblasts), which were ranked among ovary, cervix uteri and uterus as the tissues with the highest average expression levels of RPs (Figure 3). Regarding global RP expression in non-disease samples, liver and brain display the lowest RP expression levels, while ovary and cervix uteri the highest (Figure 3). Curiously, the detection of liver and blood as tissues with low RP expression levels might be due to the usage of different expression datasets and processing (50), compared to previous individual studies. Nevertheless, proliferation potential appears to account for most of the RP expression variability displayed across different tissues (50), as clearly shown by the high ranking of *in vitro* cultured EBV-lymphoblasts and fibroblasts, that differ considerably from their relative tissues of origin, blood and skin, respectively (Figure 3).

To further investigate the potential of building models that can accurately predict the tissue from RP expression patterns to assess the specificity of expression, we trained and evaluated the performance of 4 multi-classification learning models (see ‘Materials and Methods’ section). Paralog RPs with tissue-enrichment in testis were excluded from this analysis to avoid gender bias. All models performed well on predicting the type of tissue based on RP expression values alone, with the average values of all used metrics being above 90% (Table 2). Moreover, inspection of confusion matrices showed that a number of misclassified samples concern tissues of similar origin, for example breast being classified as adipose tissue samples and vice versa, suggesting that part of the learning model errors may be partly attributed to the sampling process or the choice of tissue categorization.

**Table 2.** Results from multi-classification learning model predictions of human tissues using RP expression profiles from the GTEx project (version 8) (65) (see ‘Materials and Methods’ section)

		Logistic regression		LinearSVC		SVC		Random forest	
		Arithmetic mean	Standard deviation	Arithmetic mean	Standard deviation	Arithmetic mean	Standard deviation	Arithmetic mean	Standard deviation
83 RPs	Accuracy	0.9623	0.0023	0.966	0.001	0.9697	0.0015	0.9173	0.0065
	F1 score	0.9603	0.0032	0.965	0.001	0.9697	0.0012	0.9103	0.0057
	MCC	0.9607	0.0029	0.964	0.001	0.968	0.002	0.9123	0.0065

From the 89 detected RPs in GTEx data, gender-associated RPL10L, RPS4X/eS4, RPS4Y1/2, RPL39L and RPL26L1 to avoid bias, leaving 83 RPs. All multi-classification learning models were created using scikit-learn (68).

Additionally, through inspection of pre-calculated results of GTEx data by the non-parametric method SPECS (73) (Supplementary Figure S11), we detected particular examples of several RP tissue-specific differences, including UBA52/RPL40-precursor/eL40 in blood (Supplementary Figures S11A and 12A) and RPLP1/P1 in skin and muscle (Supplementary Figures S11B and 12B). In contrast, RPs such as RPL9/uL6 (Supplementary Figures S11C and 12C) and RPS26/eS26 (Supplementary Figures S11D and 12D) lack significant deviations from the common pattern of global RP expression as counterexamples of common, non-specific tissue expression patterns.

The above findings challenge the notion of tissue-specific expression patterns of RP genes, at least in vertebrates (47–50), underlying the role of most non-paralog genes in the formation of ribosomes for their crucial role in translation, regardless of taxonomic rank and particular tissue (39,40). Our results provide a comprehensive view for the first time the contrast between sequence variability and a highly conserved, correlated mode of gene expression for most RP genes, with the few exceptions that can be seen in exceptional circumstances, mostly for paralog RPs (Supplementary Figure S12).

### Variation of RP expression is attributed to species but not tissues in the vertebrate lineage

**Figure 4.**
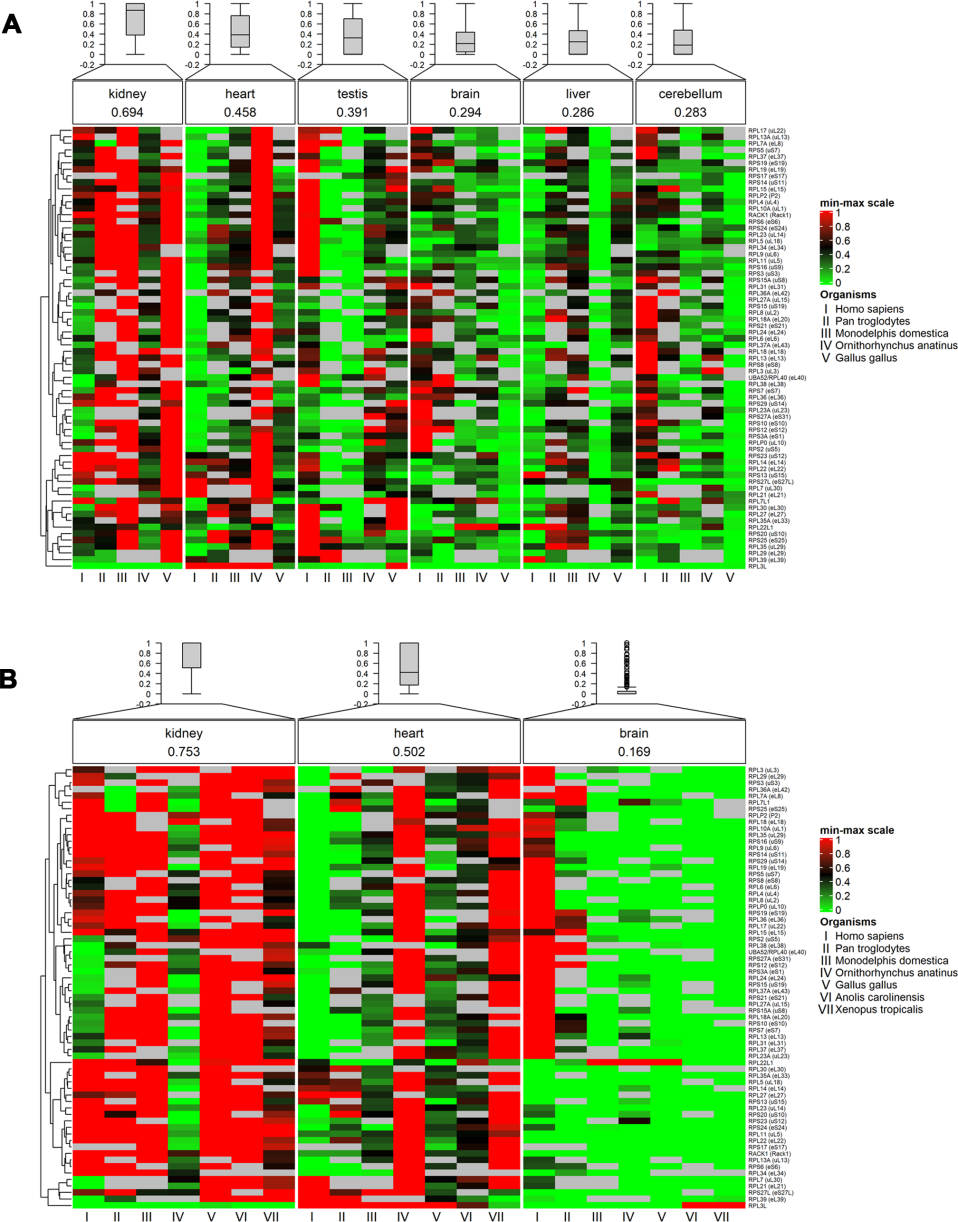
Heatmaps of expression patterns for 68 RPs across: (**A**) Six common tissues for five vertebrate species, and (**B**) three common tissues for seven vertebrate species (67) (see ‘Materials and Methods’ section). Expression values (cRPKM) were scaled between 0 and 1 using Min–Max normalization for i) each organism and ii) each RP, before being reordered per tissue for comparisons. Name, arithmetic mean and boxplot of all RP expression values are shown per tissue. Heatmaps were created using ComplexHeatmap (53).

It has been demonstrated that evolutionary analysis of gene expression patterns across various tissues of different mammals can be predictive of gene functionality and importance in disease. Specifically, genes highly conserved in both expression and sequence were shown to participate mainly in housekeeping functions, whereas those conserved in sequence but with variations in expression are primarily involved in transcriptional regulation and possibly can be attributed with species-specific differences (74). Here, we sought to investigate and characterize RP expression patterns across different vertebrate species and tissues. Initially, we utilized two combinations in which RNA-seq data of 68 RPs (corrected Reads Per Kilobase Million, cRPKM) from i) six tissues of five species, and ii) three tissues of seven species (see ‘Materials and Methods’ section), were used after scaling expression values between 0 and 1 (Min–Max scaling) for each organism and each RP (Figure 4). Despite limitations in sample availability and the presence of missing values, we observe few within-tissue differences across different species, for instance the increased levels of human RPs in cerebellum compared to, e.g. kidney, while most tissues are consistently expressed (Figure 4A). In the case of the widest possible span across species (seven in total), only three tissues present sufficient data for comparison: it is remarkable that most species (with the exception of human, to some extent) show the lowest expression of RPs in the brain, compared, e.g. to kidney (Figure 4B)—consistently with the above findings for fewer species and more tissues. Overall, there seems to be a general trend for the available instances of tissue-species combinations for higher expression in kidney and heart compared to brain and cerebellum (Figure 4). As tissues are far older biological entities than species in evolutionary terms, tissue specificity can be seen as limited, while the restricted variation observed arise from species differences.

Furthermore, in an attempt to provide an estimate of within-tissue conservation of RP expression levels across different vertebrate species, multi-classification learning models were trained using only the GTEx dataset of human tissues and evaluated for their ability to independently predict the tissue type of samples from the comparative profiling of gene expression across different species (69) (see ‘Materials and Methods’ section). Apart from lizard and frog, learning models were able to predict with significance (*P*-value < 0.1; one-tailed binomial test) the tissue type of different sample species, especially for the other three primates analyzed here (Supplementary Table S2). This pivotal result strongly suggests that tissue conservation patterns contain a stronger signal than their corresponding species counterparts, as the models are able to recognize the tissue type of different vertebrates based on human tissues, expectedly with decreasing performance. The learning models provide a quantitative perspective of the patterns reflected in the comparative analysis of tissues versus species (Figure 4), further enhancing the reliability of this key conclusion.

Overall, our data support that RP expression levels fluctuate in a consistent manner across tissues of vertebrate species. Additionally, and in agreement with previous observations of gene expression suggesting strong conservation across different tissues (69), we find evidence that human RP expression patterns enable, to a degree, an accurate prediction of tissues in different vertebrates. At the same time, we observe a limited number of within-species differences for several RPs (Supplementary Figures S11 and 12), that need to be further studied and confirmed at the protein level.

## DISCUSSION

Eukaryotic ribosomes, beyond the structurally and functionally universal ribosome core, exhibit additional complexity over their distant bacterial and affine archaeal counterparts in both rRNA and protein content. Specific RPs and other elements, some uniquely shared between archaea and eukarya, contribute interactions within the ribosome or with translation factors in domain-specific translation regulation. These features are well established and illustrate the unique evolutionary trajectories at the phylogenetic domain level (10,75). Here we present a comprehensive analysis of RP sequence conservation across eleven representative vertebrate species, the. most studied animal lineage. Detailed curation of RP protein sequences for those species has resulted in a full complement, via the identification at the nucleotide level of missing RP instances in protein sequence databases (Figure 1). Moreover, potentially systematic errors from gene prediction in Ensembl vertebrate proteomes (release 100) (54) are also complemented in our RP collection across 78 families (see ‘Results’ section). Given the universal role of RPs, these missing instances available through this work may be considered for inclusion into the above resources.

Considerable differences in sequence conservation of RPs throughout the vertebrate spectrum range from ∼69% (RPL14/eL14) to 100% (Figure 2A). Among the RPs with the highest identity are RPL38/eL38, RPS14/uS11 and RPS15a/uS8, implicated in ribosomopathies and RPL29/eL29. RPL38/eL38 is indispensable for the translation of a subset of *Hox* mRNAs (22). Similarly, heterozygous mutations in RPS14/uS11 and RPS15a/uS8 cause erythropoiesis defects in 5q- syndrome (20) and DBA (76), respectively. The functional role of RPL29/eL29 is less clear, since it is redundant for yeast survival, yet its loss causes delayed translation and is lethal only when combined with mutations in *RPL10/uL10* or *RPSA1* (77). Moreover, *RPL29/eL29* knockout results in a pathological but non-lethal murine phenotype (78), being also associated with regulation of angiogenesis (79,80).

While it is tempting to speculate that various levels of sequence conservation might suggest potentially specialized functions for some RPs, this apparent flexibility might be instrumental for the fulfilment of RP roles at the tertiary structural level (Figure 2B), as is known for three-dimensional complexes (10,81). For those five core RPs with the lowest similarity levels across vertebrates—namely: RPL14/eL14, RPL6/eL6, RPL36/eL36, FAU/RPS30-precursor/eS30 and RPL7/uL30—there is a lack of murine knockout phenotypes (82); however, they are implicated in the generation of Minute phenotypes in fruit fly (83). RPL13/eL13, at ∼80% identity between human and zebrafish presents a missense and three splice variants, the latter leading to an 18 aa insertion, with *RPL13/eL13* being the cause of a rare ribosomopathy, characterized by skeletal dysplasia (84). Furthermore, *RPL13/eL13* was discovered to be a candidate disease gene in patients with congenital heart disease, with heart-specific *RPL13/eL13* knockdowns compromising embryonal heart development in fruit fly (85).

Being some of the most important and highly conserved subset of proteins in any organism, RPs are known to be responsible for the oncogenic potential of different malignancies when mutated (86,87), while post-translational modifications may also play a significant role in that regard (88,89). Further investigation is required to shed light on why vertebrate RPs exhibit such idiosyncratic and profoundly different conservation and what is the potential significance of these sequence and length variations.

The intriguing sequence variation across RPs further dictates the examination of their expression patterns with regard to species and tissues, for non-disease states and organisms for which these data are available. Using GTEx, and in agreement with previous studies (50), we demonstrate that RP expression patterns are predominantly positively correlated, with their levels changing in a similar and consistent manner across tissues. Similar patterns are seen in cell lines and the tissues where they originate from (Figure 3). Transcriptional regulation by various factors, such as c-MYC and GATA1 (90–92), and post-transcriptional control mechanisms, such as 5′ terminal oligopyrimidine tract (5′TOP) (93) and miR-10a (94), also contribute to coordination of RP gene expression. Variations of RP gene expression in healthy tissues raise the question whether tissue-specific or extra-ribosomal functions are at play (47–50). The discovery of dynamic RP stoichiometry, by which sub-cellular populations of ribosomes are subjected to regulation by external stimuli (95) and determine the translation of selected mRNAs (95,96), strengthen this hypothesis. Nonetheless, several independent studies have not been able to identify different RP stoichiometry in proteomic analysis of isolated ribosomes (89,97–99), as well as in murine brain (hippocampus, cortex and cerebellum), liver and age groups (juvenile, adult, and middle-aged) (99). Differences may arise from paralog gene expression or limited cases in ribosomal function, as in the example of RPL3L (see *Results*) or RPLP1/P1 (Supplementary Figure S11), respectively. Our results suggest that RP gene expression exhibits limited fluctuations and those that are observed may reflect tissue idiosyncrasies associated with translation efficacy and might be independent from a coordinated ribosome function.

The most striking observation with relation to tissue-specific gene expression of RPs arises in cross-species comparisons, where most of the variation derives from species as the most evolutionarily ‘recent’ biological entities and not tissues, being more ancient (Figure 4)—consistent with findings for other processes such as splicing (69). Admittedly, there are limitations due to sample availability and missing values, yet the variation of total RP expression is less between tissues (Figure 4A) and attributable more to species variation (Figure 4B), strongly supported by the machine learning models (see ‘Results’ section).

Despite limitations in sample availability and the presence of missing values, we observe few within-tissue differences across different species, for instance the increased levels of human RPs in cerebellum compared to, e.g. kidney, while most tissues are consistently expressed (Figure 4A). In the case of the widest possible span across species (seven in total), only three tissues present sufficient data for comparison: it is remarkable that most species (with the exception of human, to some extent) show the lowest expression of RPs in the brain, compared, e.g. to kidney (Figure 4B)—consistently with the above findings for fewer species and more tissues. Overall, there seems to be a general trend for the available instances of tissue-species combinations for higher expression in kidney and heart compared to brain and cerebellum (Figure 4). As tissues are far older biological entities than species in evolutionary terms, tissue specificity can be seen as limited, while the restricted variation observed arise from species differences. One reasonable interpretation for the patterns of discordant sequence conservation and transcript abundance is a lineage-specific trajectory that is determined by differences from evolutionary change and adaptation (Supplementary Figure S14), while maintaining a complex, coordinated process for the structure and function of ribosomes in their fundamental role in translation. The peculiar, antagonistic functions of RP paralogs, as distinct exceptions, suggest that co-regulated and consistent RP expression levels serve to maintain exact ribosome counts for some tissues and mediate effective translation for selected mRNA groups.

## DATA AVAILABILITY

Files of the entire RP sequence collection, the curated entries and all gene expression information in this study as well as scripts (shell/R/python) used for analysis are available on Figshare https://figshare.com/projects/VRP-vertebrate_ribosomal_proteins/83864.

## Supplementary Material

lqaa088_Supplemental_Files
