# Supplementary material for: Sequence variation, common tissue expression patterns and learning models: a genome-wide survey of vertebrate ribosomal proteins
Source: NAR Genom Bioinform. 2020 Nov 6;2(4):lqaa088. doi: 10.1093/nargab/lqaa088 (PMC7671327; doi:10.1093/nargab/lqaa088)
Supplement: lqaa088_Supplemental_Files [file lqaa088_supplemental_files.zip › Supplementary_Data_revised_v2.docx]

Supplementary Data for

*Sequence variation, common tissue expression patterns and learning models: a genome-wide survey of vertebrate ribosomal proteins*

Konstantinos A. Kyritsis^1,2^, Christos A. Ouzounis^2*^, Lefteris Angelis^3^, Ioannis S. Vizirianakis^1,4,5*^

^1^Laboratory of Pharmacology, School of Pharmacy, Aristotle University of Thessaloniki, GR-54124 Thessalonica, Greece

^2^Biological Computation & Process Laboratory, Chemical Process & Energy Resources Institute, Centre for Research & Technology Hellas, GR-57001 Thessalonica, Greece

^3^Department of Informatics, Aristotle University of Thessaloniki, GR-54124 Thessalonica, Greece

^4^FunPATH (Functional Proteomics and Systems Biology Research Group at AUTH) Research Group, KEDEK - Aristotle University of Thessaloniki, Balkan Center, GR-57001, Thessalonica, Greece

^5^Department of Life and Health Sciences, University of Nicosia, CY-1700 Nicosia, Cyprus

^*^correspondence: ISV ivizir@pharm.auth.gr or CAO ouzounis@certh.gr

Keywords: ribosomal proteins, tissue-specific gene expression, GTEx, vertebrate lineage

Files of the entire ribosomal protein sequence collection, the curated entries and all gene expression information in this study as well as scripts (shell/R/python) used for analysis, are available on Figshare:

Abbreviations:

Ribosomal Proteins: (RPs)

Multiple Sequence Alignment: (MSA)

t-Distributed Stochastic Neighbor Embedding: (t-SNE)

List of Supplementary Tables

**Supplementary Table S1.** RPs missing BLASTP hits in vertebrate proteomes.

3

**Supplementary Table S2.** Multi-classification learning model predictions of tissues for ten representative vertebrate species. 4

List of Supplementary Figures

**Supplementary Figure S1.** Differences in sequence length between Homo sapiens and Danio rerio ortholog RPs. 6

**Supplementary Figure S2.** MSA for RPL29/eL29 ortholog sequences of eleven vertebrate species.

7

**Supplementary Figure S3.** MSA for RPL14/eL14 ortholog sequences of eleven vertebrate species.

7

**Supplementary Figure S4.** MSA for RPL4/uL4 ortholog sequences from eleven vertebrate species.

. 8

**Supplementary Figure S5.** RP paralog pairs expression profiles for human tissues.

9

**Supplementary Figure S6.** Two-dimension reduction based on RP expression values of 33 human tissues using t-SNE (*see Methods*; separate HTML file).

**Supplementary Figure S7.** Three-dimension reduction based on RP expression values of 33 human tissues using t-SNE (*see Methods*; separate HTML file).

**Supplementary Figure S8.** Heatmap of RP expression profiles in human tissues with low number of samples. 10

**Supplementary Figure S9.** Heatmap of RP expression profiles in human tissues with moderate number of samples. 11

**Supplementary Figure S10.** Heatmap of RP expression profiles in human tissues with high number of samples. 12

**Supplementary Figure S11.** Expression profiles of tissue-enriched UBA52/RPL40-precursor/eL40 and RPLP1/P1, as well as constitutively expressed RPL9/uL6 and RPS26/eS26, across human tissues, based on the non-parametric method SPECS.

13

**Supplementary Figure S12.** Expression profiles of tissue-enriched UBA52/RPL40-precursor/eL40 and RPLP1/P1, as well as constitutively expressed RPL9/uL6 and RPS26/eS26, across human tissues.

14

**Supplementary Figure S13.** Correlation patterns of RP expression profiles in human tissues.

15

**Supplementary Figure S14.** Relationship between RP sequence conservation and human tissue expression profiles.

16

**Taxon-specific Expansion Segments in Vertebrates**

Although RPs exhibit almost identical size and amino acid composition within each domain of life (Bacteria, Archaea and Eukarya), unique sequence features, such as decorations, extensions and deletions, exist across evolutionarily distant species within the same domain, which have been proposed to illustrate functional adaptations of life to different environments (1–3). We compared the size of the largest protein sequences (our code: ref_0) between human and zebrafish RP orthologs and identified 8 human RPs with over 20 additional amino acid (aa) residues compared to their zebrafish orthologs, while human RPSA/uS2 is shorter than its zebrafish ortholog by 8 aa (**Supplementary Figure S1**). The difference of 158 aa between the human RPS24/eS24 and its zebrafish counterpart concerns only one of its isoforms (NP_001135757.1), while the rest show similar length with the zebrafish isoform 1 (NP_001012316.1) (~130 aa). Additionally, a RPL36a/eL42 difference of 36 aa concerns an outdated human sequence version (NP_066357.2) which has been updated (NP_066357.3) and now exhibits equal length with its zebrafish ortholog (NP_001189439.1) (106 aa). Therefore, in order to avoid ambivalence, we opted to focus on three RPs, RPL29/eL29, RPL14/eL14 and RPL4/uL4, that show significant ortholog length differences (>50 aa), while not concerning specific isoforms only. We performed MSA for the orthologs of RPL29/eL29, RPL14/eL14 and RPL4/uL4, using the sequences from our curated RP collection (**Supplementary Figures S2-S4**) (see *Methods*). We observed that the N-terminal part of all three RPs is highly conserved and consists of RP-specific superfamily domains, which include a) Ribosomal_L29e for RPL29/eL29, b) Ribosomal_L14e and RPL14_KOW for RPL14/eL14, and c) PTZ00428 (60S ribosomal protein L4) for RPL4/uL4. Size differences between human and zebrafish RPs are caused by the presence of C-terminal Expansions Segments (CES), whose length and sequence varies considerably across vertebrate taxonomic groups, with mammalian sequences being the longest.

For human RPL29/eL29 (159 aa) the difference between the conserved N- and the varying CES is pronounced (95 aa), with its zebrafish ortholog (64 aa) being almost entirely comprised of the conserved region (53 aa) (**Supplementary Figure S2**). Conservation of the first ~50 aa in RPL29/eL29 has been previously noted between orthologs of various eukaryotes that include mouse, zebrafish and fruit fly (4, 5). Notably, both human and murine RPL29/eL29 were identified as heparin/heparan sulfate (Hp/HS) interacting proteins that modulate cell adhesion and blood coagulation (6, 7). Additionally, solid-phase and affinity chromatography assays of human and murine recombinant RPL29/eL29 *in vitro*, revealed that truncated forms of this RP, missing either N- or C-terminal parts, retain their ability to bind Hp/HS albeit with reduced affinity when compared to the intact protein. These findings support that RPL29/eL29 has extra-ribosomal roles through its affinity with Hp/HS, which is mediated by multiple domains across the entire protein (5). The reduced but significant Hp/HS affinity of the truncated human RPL29/eL29 (with 79 aa missing from its C-terminus) leads to the hypothesis that the conserved zebrafish ortholog may also be capable of binding Hp/Hs and that the taxon-specific C-terminal ES might constitute an evolutionary adaptation that confers increased Hp/Hs binding capability to mammals.

For RPL14/eL14 and RPL4/uL4, studies in yeast models have illustrated the importance of their eukaryotic-specific CES in ribosome biogenesis. In particular, 5-FOA yeast plasmid shuffling assays have shown that truncated *rpl14/eL14* mutants, lacking either 29 aa (one alpha-helix) or 41 aa (both alpha-helices) from their C-termini, had impaired growth, probably to due lack of interaction with RPL13a/uL13 (yeast ortholog RPL16) C-terminus. In addition, synergistic lethal effects were observed in yeast upon combination of truncated *rpl14/eL14* mutant (with 16 aa loss from its C-terminus) with mutants for the trans-acting factors *mak5*, *ebp2* and *nop16* (8). Similarly, loss of CES for RPL4/uL4 in yeast resulted in impaired growth. It was further demonstrated that RPL4/uL4 C-terminal extension harbors a Nuclear Localization Signal (NLS), that facilitates interaction with karyopherin Kap104 and nuclear translocation of the RPL4/uL4 in complex with its chaperone, Acl4. Additionally, RPL4/uL4 CES was also required for the release of Acl4 and the incorporation of RPL4/uL4 into the LSU (9).

Despite these findings, yeast RPL14/eL14 and RPL4/uL4 CES are shorter than those of higher eukaryotes, and the observed discrepancies in CES size across different vertebrate taxa are still puzzling. Interestingly, RPL4/uL4 CES has been previously reported to be well-conserved in human, mouse (*Mus musculus*) and rat (*Rattus norvegicus*) but not in frog (*Xenopus tropicalis*) or zebrafish (10). Like many other RPs who have been associated with viral functionality, RPL4/uL4 was found to be necessary for the DNA episome maintenance and transcription activation mediated by Epstein–Barr Virus (EBV) Nuclear Antigen 1 (EBNA1) *in vitro*. Specifically, while the conserved RPL4/uL4 N-terminus is required for the RP’s EBNA1-induced nuclear translocation, C-terminal lysine (K) residues K380 and K393 are essential for the histone H3 lysine 4 dimethylation (H3K4me2) modification of episome DNA and the subsequent EBNA1 activation of transcription (10). This Hp/Hs binding and EBV exploitation of the RPL29/eL29 and RPL4/uL4 CES, respectively, support underlying functional roles for these sequences, and suggest the same for mammalian RPL14/eL14 CES.

Supplementary Tables

**Supplementary Table S1.** Number of sequences (233 reported; 210 unique) for each of the 19 RPs, missing BLASTP hits (e-value<0.05) in vertebrate proteomes (see **Methods**). Total proteomes for the eleven vertebrate species were retrieved from Ensembl (release 100) (17).

| Ensembl Proteomes (release 100) | RPL10/ uL16 | RPL10L | RPL13/ eL13 | RPL13a/ uL13 | RPL18/ eL18 | RPL27a/ uL15 | RPL28/ eL28 | RPL29/ eL29 | RPL34/ eL34 | RPL36/ eL36 |
| --- | --- | --- | --- | --- | --- | --- | --- | --- | --- | --- |
| *Anolis carolinensis* | 0 | 0 | 0 | 0 | 0 | 0 | 0 | 0 | 11 | 11 |
| *Danio rerio* | 0 | 0 | 0 | 0 | 0 | 12 | 0 | 0 | 0 | 0 |
| *Gallus gallus* | 14 | 8 | 0 | 12 | 12 | 0 | 18 | 0 | 0 | 0 |
| *Homo sapiens* | 0 | 0 | 0 | 0 | 0 | 0 | 0 | 0 | 0 | 0 |
| *Macaca mulatta* | 0 | 0 | 0 | 0 | 0 | 0 | 0 | 0 | 0 | 0 |
| *Monodelphis domestica* | 0 | 0 | 0 | 0 | 0 | 0 | 0 | 0 | 0 | 0 |
| *Mus musculus* | 0 | 0 | 0 | 0 | 0 | 0 | 0 | 0 | 0 | 0 |
| *Ornithorhynchus anatinus* | 0 | 0 | 0 | 0 | 0 | 0 | 0 | 12 | 0 | 0 |
| *Pan troglodytes* | 0 | 0 | 0 | 0 | 0 | 0 | 0 | 0 | 0 | 0 |
| *Pongo abelii* | 0 | 0 | 13 | 0 | 0 | 0 | 0 | 0 | 0 | 0 |
| *Xenopus tropicalis* | 0 | 0 | 0 | 0 | 0 | 0 | 0 | 0 | 0 | 0 |
|  |  |  |  |  |  |  |  |  |  |  |
| Ensembl Proteomes (release 100) | **RPL39L** | **RPLP2/ P2** | **RPS18/ uS13** | **RPS19/ eS19** | **RPS20/ uS10** | **RPS28/ eS28** | **RPS29/ uS14** | **RPS5/ uS7** | **RPS9/ uS4** |  |
| *Anolis carolinensis* | 1 | 0 | 0 | 12 | 12 | 11 | 13 | 0 | 0 |  |
| *Danio rerio* | 1 | 0 | 0 | 0 | 12 | 0 | 0 | 0 | 0 |  |
| *Gallus gallus* | 1 | 0 | 12 | 0 | 0 | 0 | 0 | 11 | 3 |  |
| *Homo sapiens* | 0 | 0 | 0 | 0 | 0 | 0 | 0 | 0 | 0 |  |
| *Macaca mulatta* | 2 | 0 | 0 | 0 | 0 | 0 | 0 | 0 | 0 |  |
| *Monodelphis domestica* | 1 | 0 | 0 | 0 | 0 | 0 | 0 | 1 | 0 |  |
| *Mus musculus* | 1 | 0 | 0 | 0 | 0 | 0 | 0 | 0 | 0 |  |
| *Ornithorhynchus anatinus* | 1 | 12 | 0 | 0 | 0 | 0 | 0 | 0 | 0 |  |
| *Pan troglodytes* | 0 | 0 | 0 | 0 | 0 | 0 | 0 | 0 | 0 |  |
| *Pongo abelii* | 1 | 0 | 0 | 0 | 0 | 0 | 0 | 0 | 0 |  |
| *Xenopus tropicalis* | 1 | 0 | 0 | 0 | 0 | 0 | 0 | 0 | 1 |  |

**Supplementary Table S2.** Results from multi-classification learning model predictions of tissues for ten representative vertebrate species (see ***Methods***) (18). Due to limitations of available RP expression values and tissues, the number of RP expression profiles and tissues available for each vertebrate species, were extracted from GTEx (shown here for each species) (19). Vertebrate species, for which learning model(s) perform with greater than random accuracy, are marked (p-value<0.05 (‡); p-value<0.1 (†); one-tailed binomial test).

| **Ribosomal proteins** | **Tissues** | Species | **Metric** | **Logistic Regression** | **LinearSVC** | **SVC** | **Random Forest** |
| --- | --- | --- | --- | --- | --- | --- | --- |
| 78 | 7 | *^‡^Homo sapiens* | Accuracy | 0,857 | 0,857 | 0,571 | 0,714 |
|  |  |  | Binomial test of accuracy | 5,22E-05 | 5,22E-05 | 1,02E-02 | 9,70E-04 |
|  |  |  | F1 score | 0,81 | 0,81 | 0,452 | 0,643 |
|  |  |  | MCC | 0,854 | 0,854 | 0,556 | 0,72 |
|  |  |  |  |  |  |  |  |
| 52 | 6 | *^‡^Pan troglodytes* | Accuracy | 0,833 | 0,5 | 0,833 | 0,667 |
|  |  |  | Binomial test of accuracy | 0,0007 | 0,0623 | 0,0007 | 0,0087 |
|  |  |  | F1 score | 0,778 | 0,361 | 0,778 | 0,556 |
|  |  |  | MCC | 0,828 | 0,467 | 0,828 | 0,645 |
|  |  |  |  |  |  |  |  |
| 12 | 5 | *^‡^Pongo abelii* | Accuracy | 0,8 | 0,6 | 0,6 | 0,8 |
|  |  |  | Binomial test of accuracy | 0,0067 | 0,0579 | 0,0579 | 0,0067 |
|  |  |  | F1 score | 0,733 | 0,467 | 0,467 | 0,733 |
|  |  |  | MCC | 0,791 | 0,559 | 0,559 | 0,791 |
|  |  |  |  |  |  |  |  |
| 17 | 6 | *^†^Macaca mulatta* | Accuracy | 0,5 | 0,5 | 0,167 | 0,5 |
|  |  |  | Binomial test of accuracy | 0,0623 | 0,0623 | 0,6651 | 0,0623 |
|  |  |  | F1 score | 0,5 | 0,444 | 0,056 | 0,5 |
|  |  |  | MCC | 0,414 | 0,43 | 0 | 0,414 |
|  |  |  |  |  |  |  |  |
| 30 | 7 | *^‡^Mus musculus* | Accuracy | 0,714 | 0,429 | 0,714 | 0,286 |
|  |  |  | Binomial test of accuracy | 0,0010 | 0,0652 | 0,0010 | 0,2635 |
|  |  |  | F1 score | 0,667 | 0,381 | 0,643 | 0,167 |
|  |  |  | MCC | 0,683 | 0,35 | 0,72 | 0,185 |
|  |  |  |  |  |  |  |  |
| 53 | 6 | *^†^Monodelphis domestica* | Accuracy | 0,333 | 0,5 | 0,5 | 0,5 |
|  |  |  | Binomial test of accuracy | 0,2632 | 0,0623 | 0,0623 | 0,0623 |
|  |  |  | F1 score | 0,278 | 0,444 | 0,417 | 0,361 |
|  |  |  | MCC | 0,215 | 0,414 | 0,447 | 0,467 |
|  |  |  |  |  |  |  |  |
| 56 | 6 | *^†^Ornithorhynchus anatinus* | Accuracy | 0,333 | 0,5 | 0,333 | 0,5 |
|  |  |  | Binomial test of accuracy | 0,2632 | 0,0623 | 0,2632 | 0,0623 |
|  |  |  | F1 score | 0,333 | 0,444 | 0,25 | 0,389 |
|  |  |  | MCC | 0,207 | 0,414 | 0,224 | 0,43 |
|  |  |  |  |  |  |  |  |
| 62 | 7 | *^†^Gallus gallus* | Accuracy | 0,429 | 0,286 | 0,286 | 0,429 |
|  |  |  | Binomial test of accuracy | 0,0652 | 0,2635 | 0,2635 | 0,0652 |
|  |  |  | F1 score | 0,286 | 0,19 | 0,214 | 0,286 |
|  |  |  | MCC | 0,36 | 0,175 | 0,18 | 0,36 |
|  |  |  |  |  |  |  |  |
| 55 | 4 | *Anolis carolinensis* | Accuracy | 0,25 | 0,25 | 0,5 | 0,25 |
|  |  |  | Binomial test of accuracy | 0,6836 | 0,6836 | 0,2617 | 0,6836 |
|  |  |  | F1 score | 0,167 | 0,167 | 0,333 | 0,167 |
|  |  |  | MCC | 0 | 0 | 0,408 | 0 |
|  |  |  |  |  |  |  |  |
| 63 | 5 | *Xenopus tropicalis* | Accuracy | 0,4 | 0,4 | 0,4 | 0,2 |
|  |  |  | Binomial test of accuracy | 0,2627 | 0,2627 | 0,2627 | 0,6723 |
|  |  |  | F1 score | 0,267 | 0,267 | 0,3 | 0,1 |
|  |  |  | MCC | 0,28 | 0,28 | 0,299 | 0 |


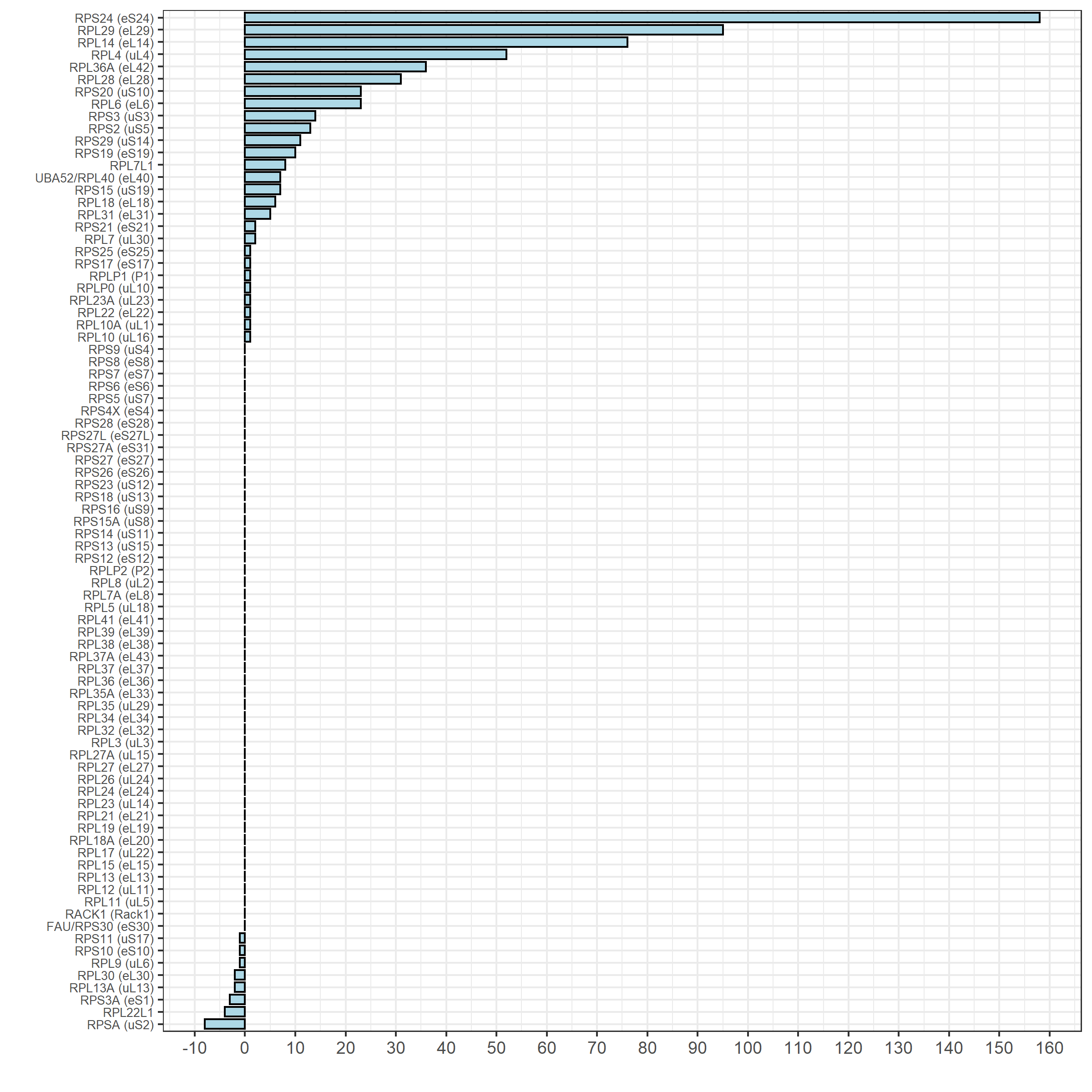
Supplementary Figures

**Supplementary Figure S1.** Differences in RP sequence length (x-axis) between Homo sapiens (human) and Danio rerio (zebrafish) ortholog RPs (y-axis) (see **Results**). Isoforms of RP orthologs with the largest size (ref_0) from both species were used for the comparisons.


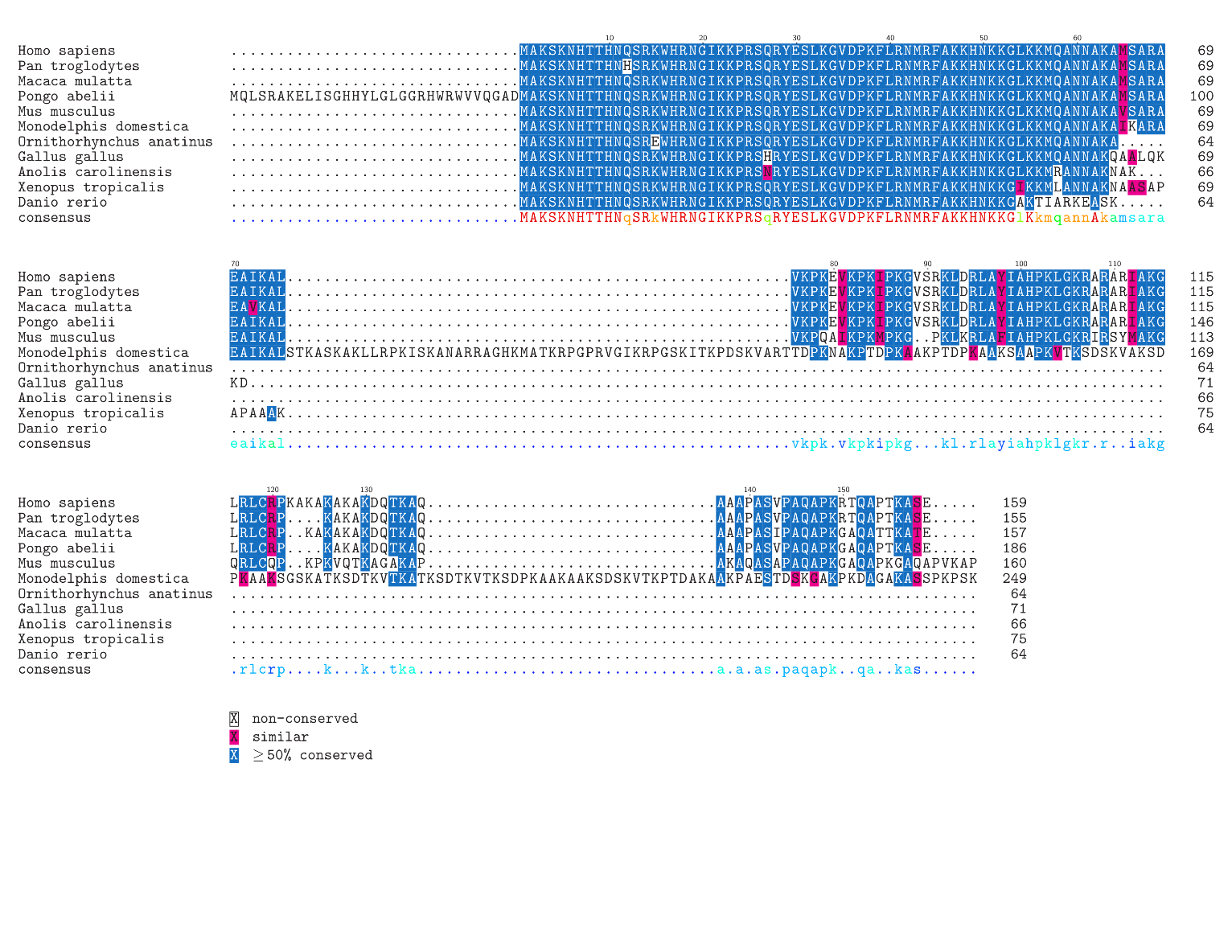

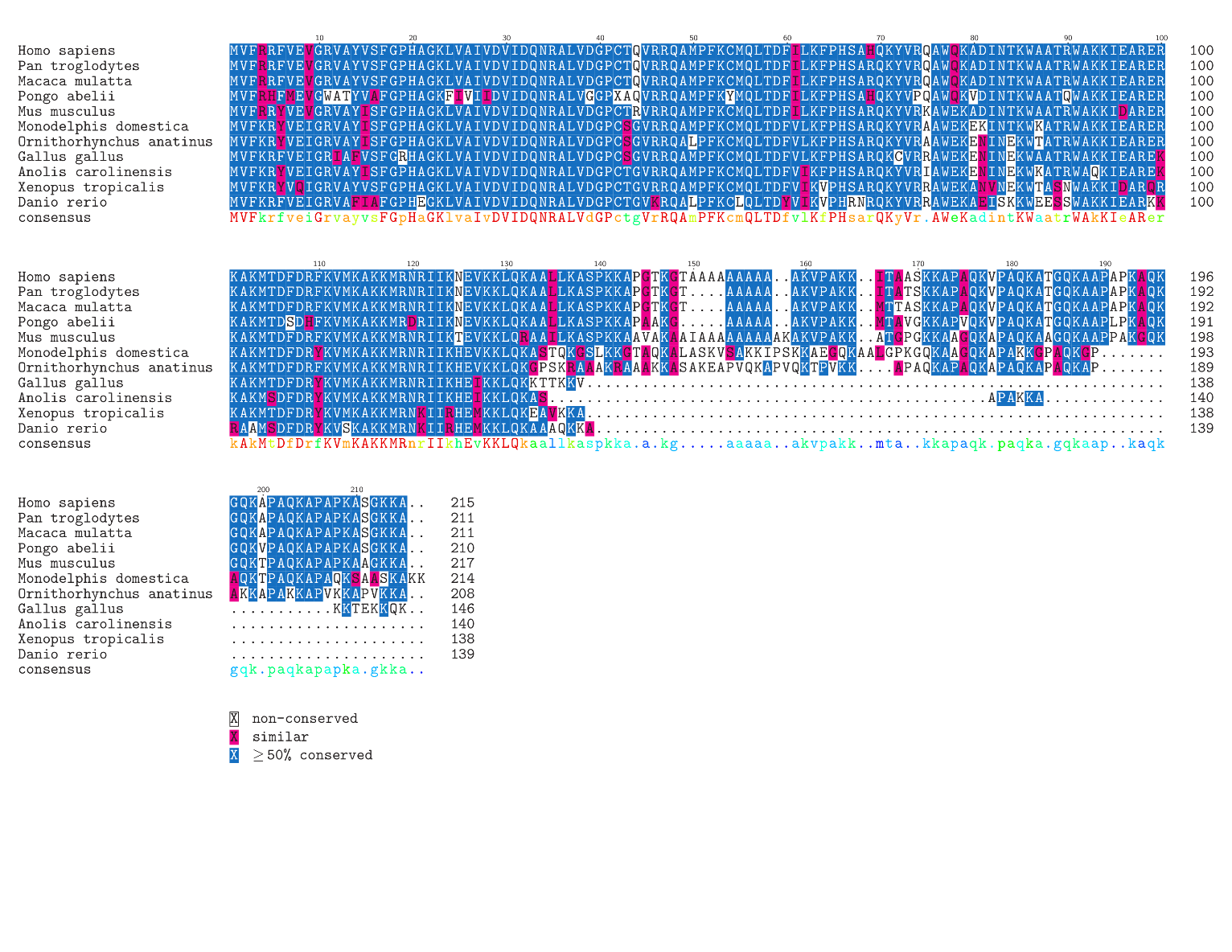


**Supplementary Figure S2.** MSA for RPL29/eL29 ortholog sequences of eleven vertebrate species. Ortholog RP sequences were retrieved from our RP collection and aligned using MUSCLE (11) and msa (12).

**Supplementary Figure S3.** MSA for RPL14/eL14 ortholog sequences of eleven vertebrate species. Ortholog RP sequences were retrieved from our RP collection and aligned using MUSCLE (11) and msa (12).

**Supplementary Figure S4.** MSA for RPL4/uL4 ortholog sequences from eleven vertebrate species. Ortholog RP sequences were retrieved from our RP collection and aligned using MUSCLE (11) and msa (12).


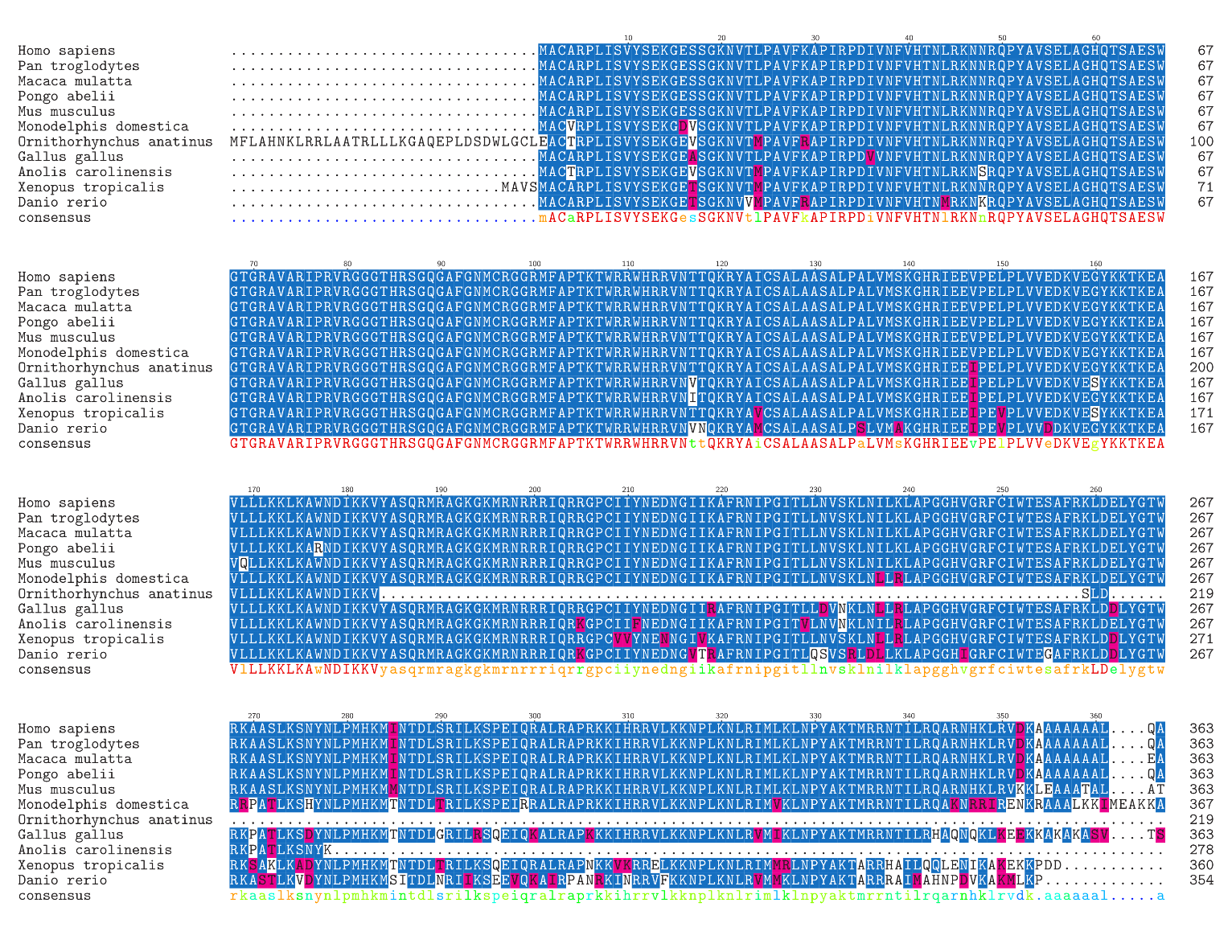

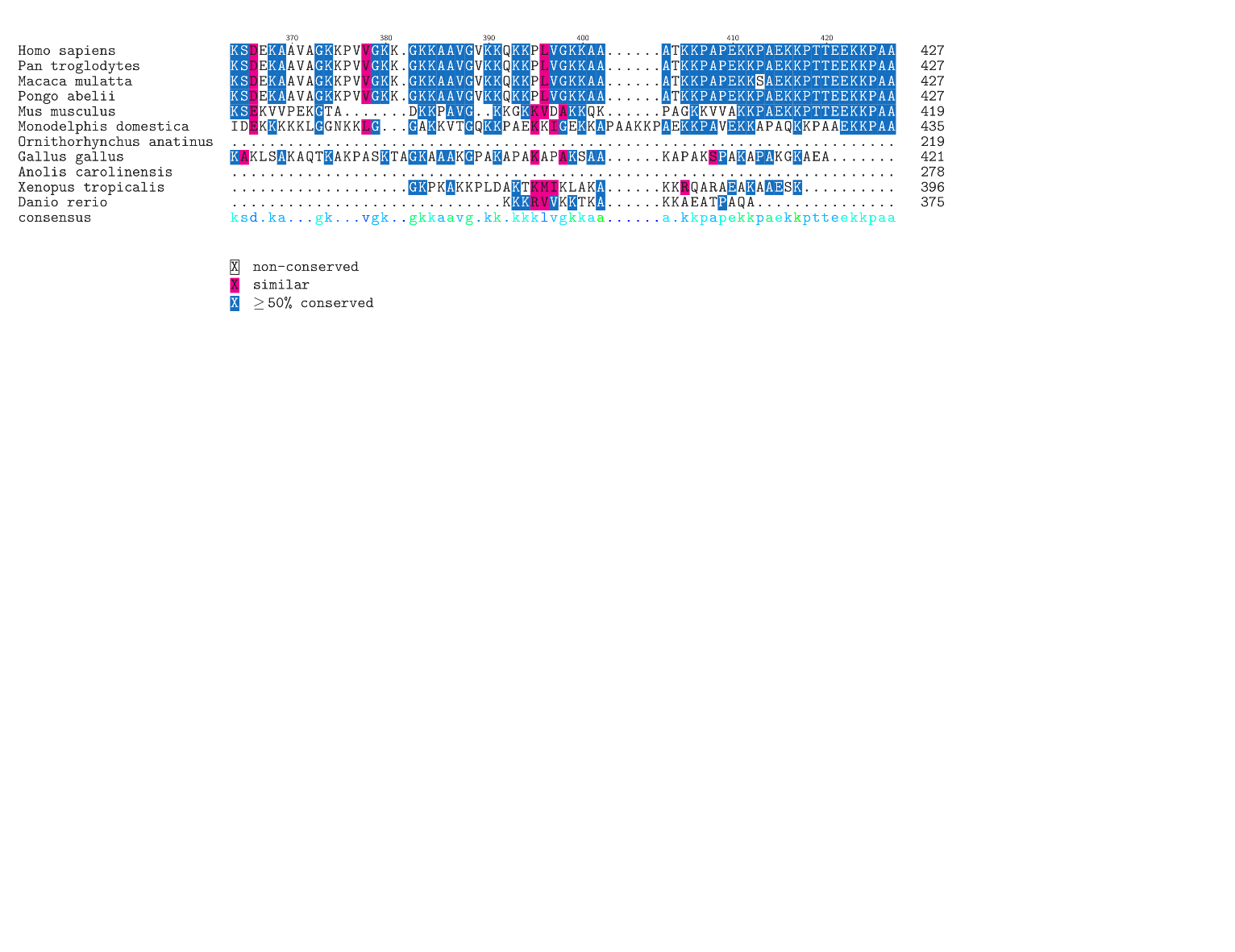


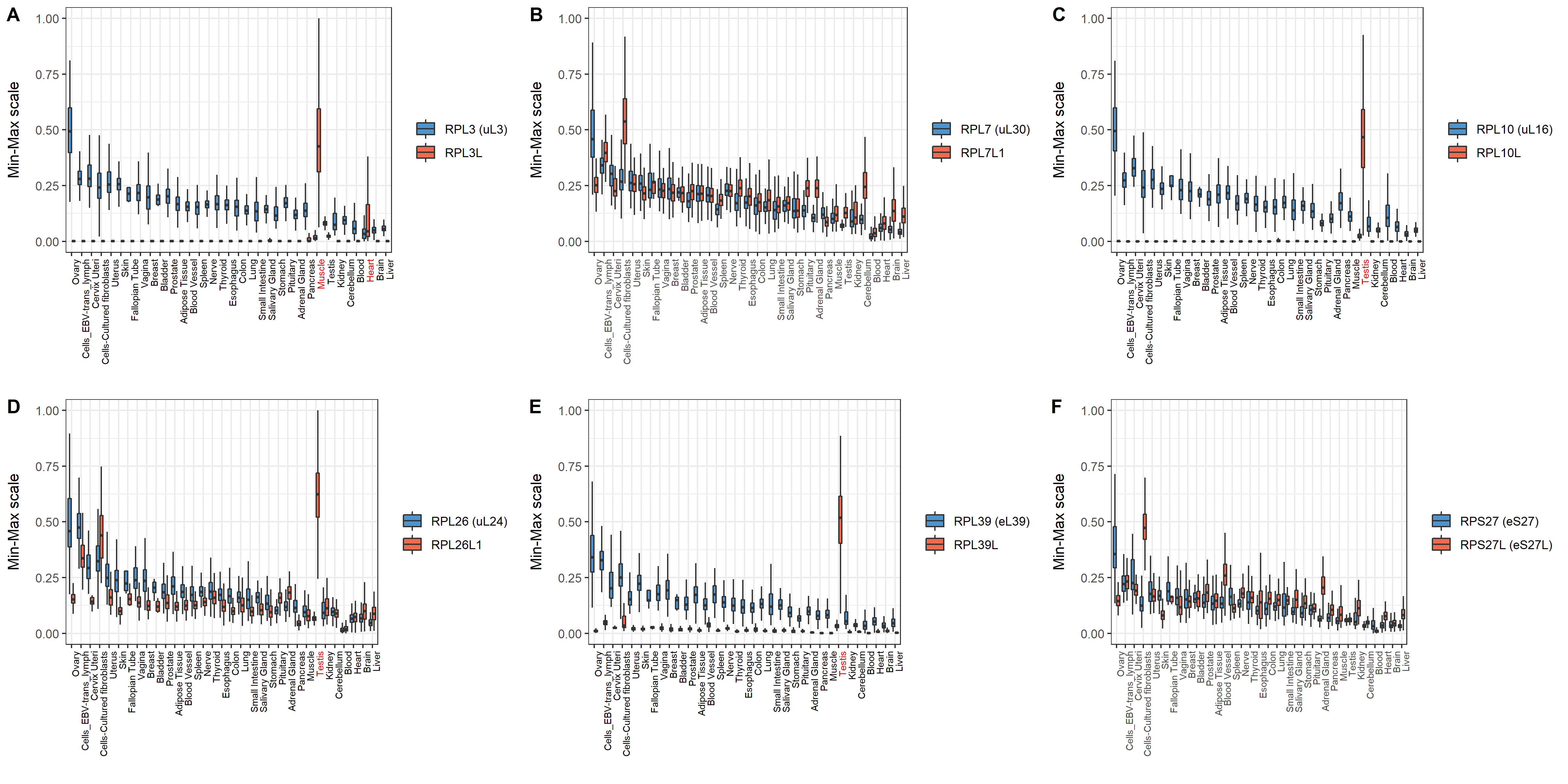


**Supplementary Figure S5.** Boxplots of RP paralog pairs expression profiles (TPM) for human tissues of the GTEx project (19). Expression values (y-axis) were scaled between 0 and 1 using Min-Max normalization. Tissue names (x-axis) with enriched RP paralog expression are highlighted in red. RP paralog pairs shown here are: (**A**) RPL3L and RPL3/uL3, (**B**) RPL7L1 and RPL7/uL30, (**C**) RPL10L and RPL10/uL16, (**D**) RPL26L1 and RPL26/uL24, (**E**) RPL39L and RPL39/eL39 and (**F**) RPS27L/eS27L and RPS27/eS27.


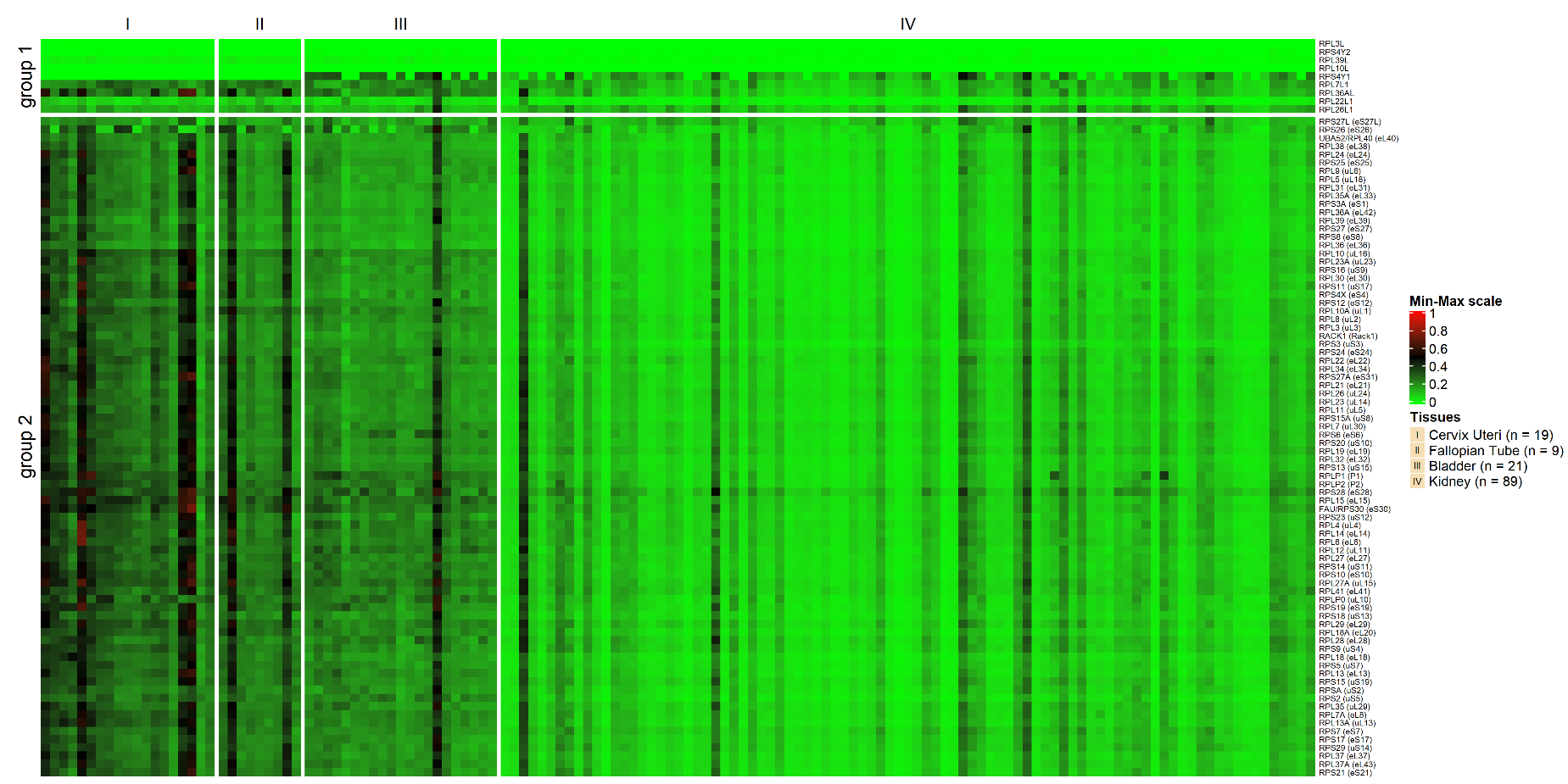


**Supplementary Figure S8.** Heatmap of 89 RP expression profiles (TPM) in human tissues of GTEx project (19), with low number of samples (less than 100 per tissue). Expression values were scaled between 0 and 1 using Min-Max normalization. RPs were ordered based on hierarchical clustering (complete agglomeration method) performed on all human tissues of the GTEx expression dataset. Paralog (group 1) and core (group 2) RPs are shown in separate heatmap blocks. Heatmap was created with ComplexHeatmap (20).


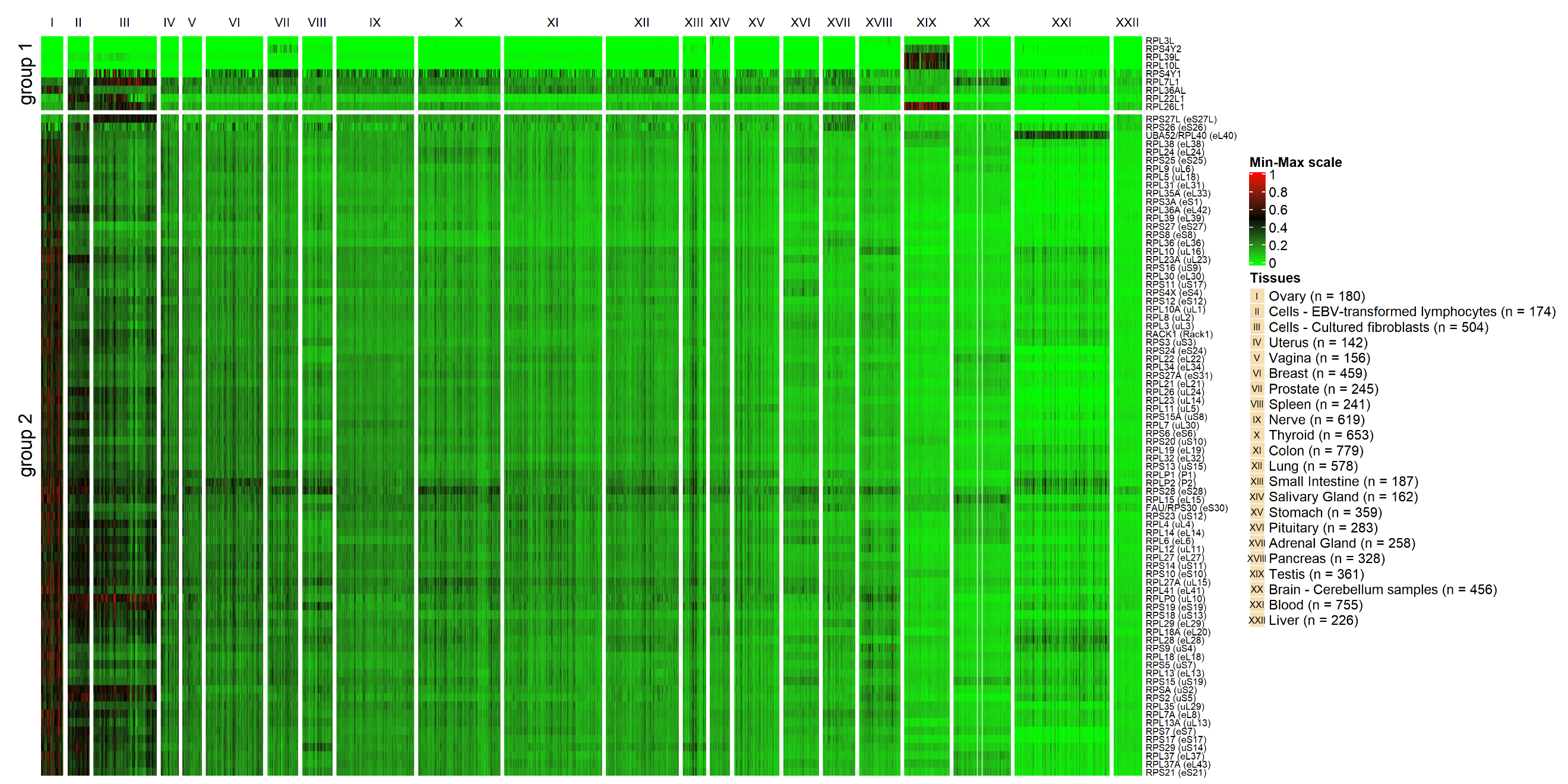


**Supplementary Figure S9.** Heatmap of 89 RP expression profiles (TPM) in human tissues of GTEx project (19), with moderate number of samples (less than 800 per tissue). Expression values were scaled between 0 and 1 using Min-Max normalization. RPs were ordered based on hierarchical clustering (complete agglomeration method) performed on all human tissues of the GTEx expression dataset. Paralog (group 1) and core (group 2) RPs are shown in separate heatmap blocks. Heatmap was created with ComplexHeatmap (20).


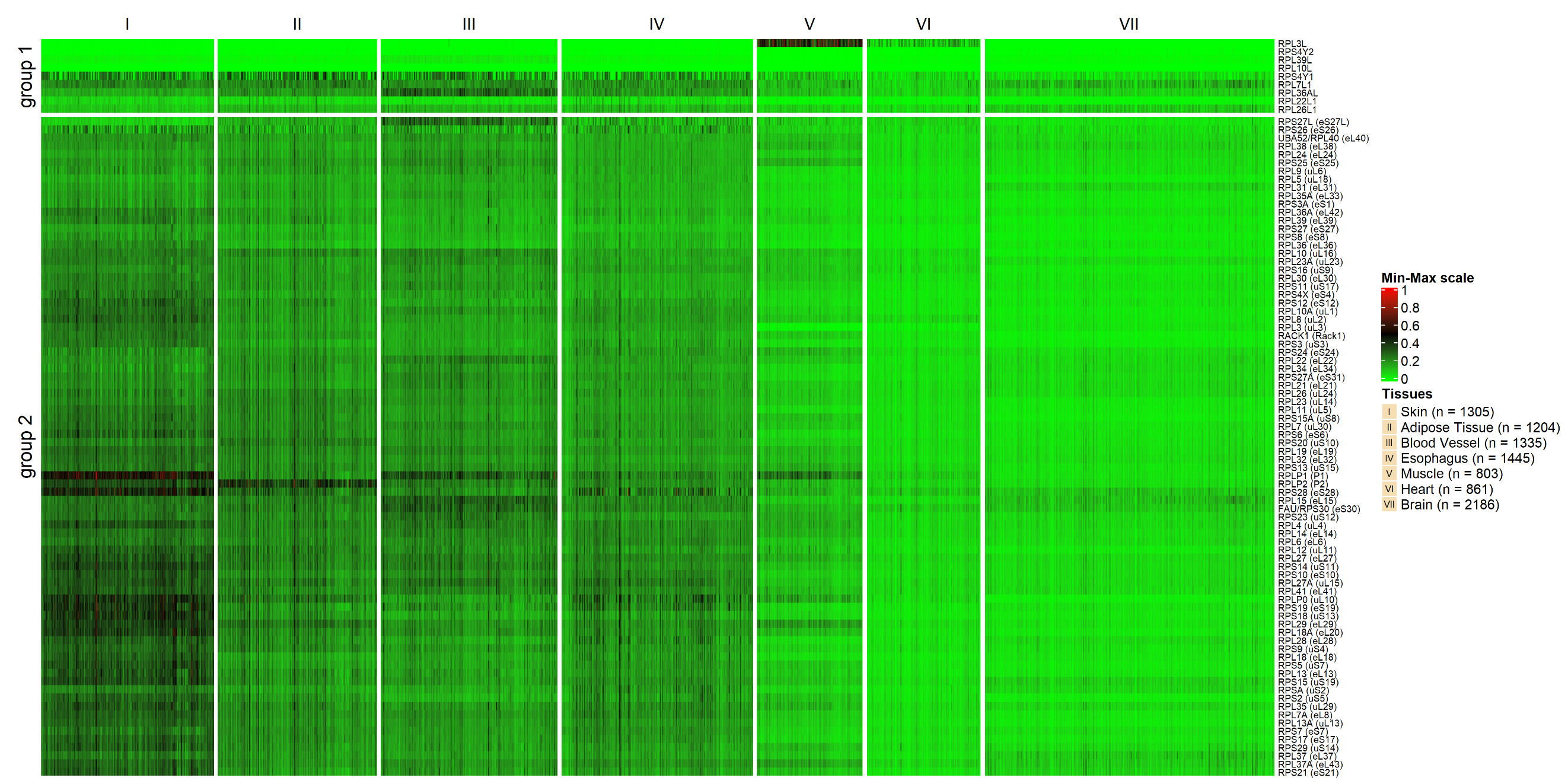


**Supplementary Figure S10.** Heatmap of 89 RP expression profiles (TPM) in human tissues of GTEx project (19), with high number of samples (more than 800 per tissue). Expression values were scaled between 0 and 1 using Min-Max normalization. RPs are ordered based on hierarchical clustering (complete agglomeration method) performed on all human tissues of the GTEx expression dataset. Paralog (group 1) and core (group 2) RPs are shown in separate heatmap blocks. Heatmap was created with ComplexHeatmap (20).


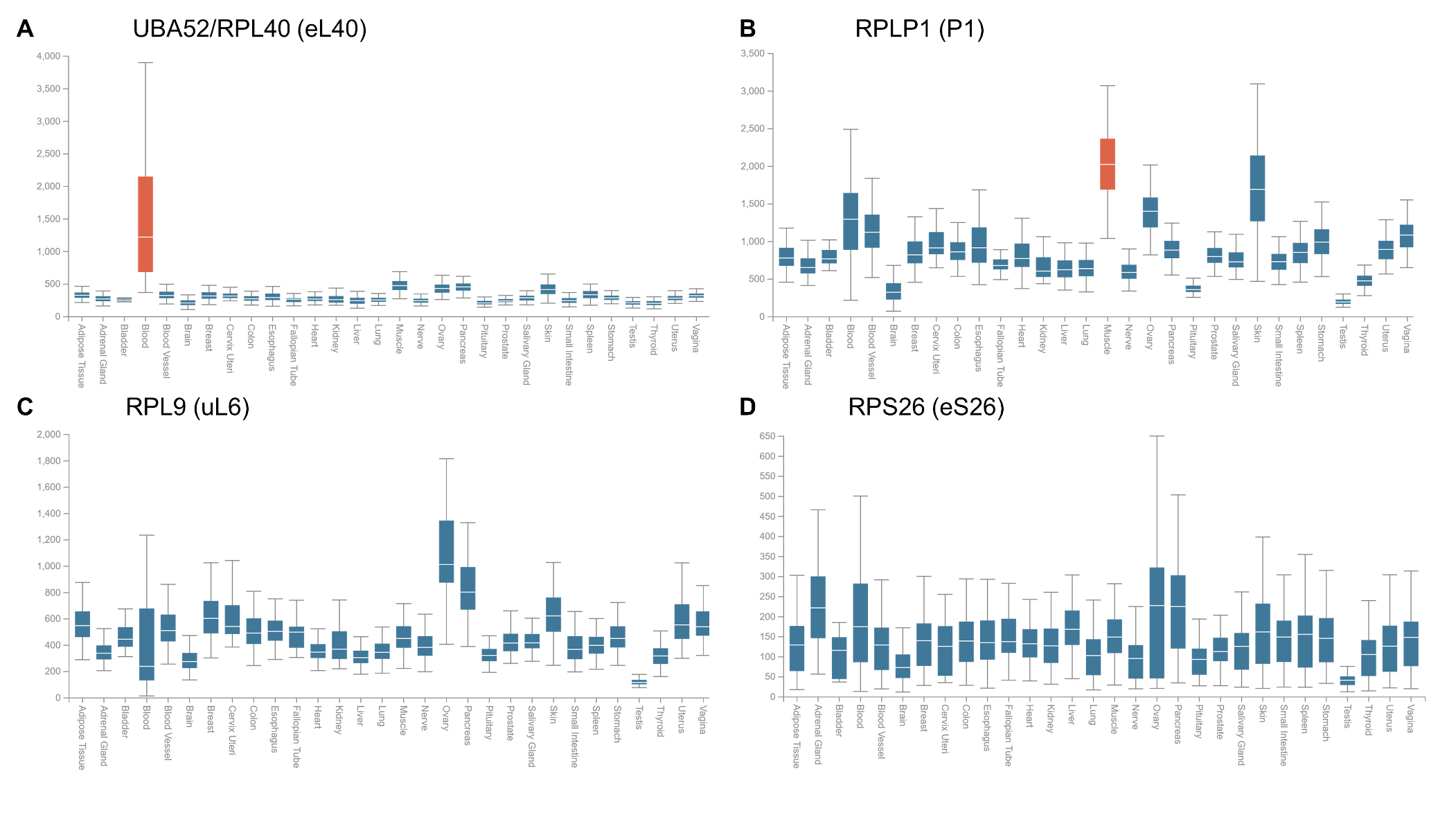


**Supplementary Figure S11.** Boxplots of tissue-enriched (**A**) UBA52/RPL40-precursor/eL40 (enriched in blood) and (**B**) RPLP1/P1 (enriched in muscle and skin), as well as constitutively expressed (**C**) RPL9/uL6 and (**D**) RPS26/eS26, presenting their expression profiles across the human tissues of GTEx project (19). Tissue-specificity was pre-determined by the non-parametric method SPECS and figures were retrieved from the respective browser tool (21).


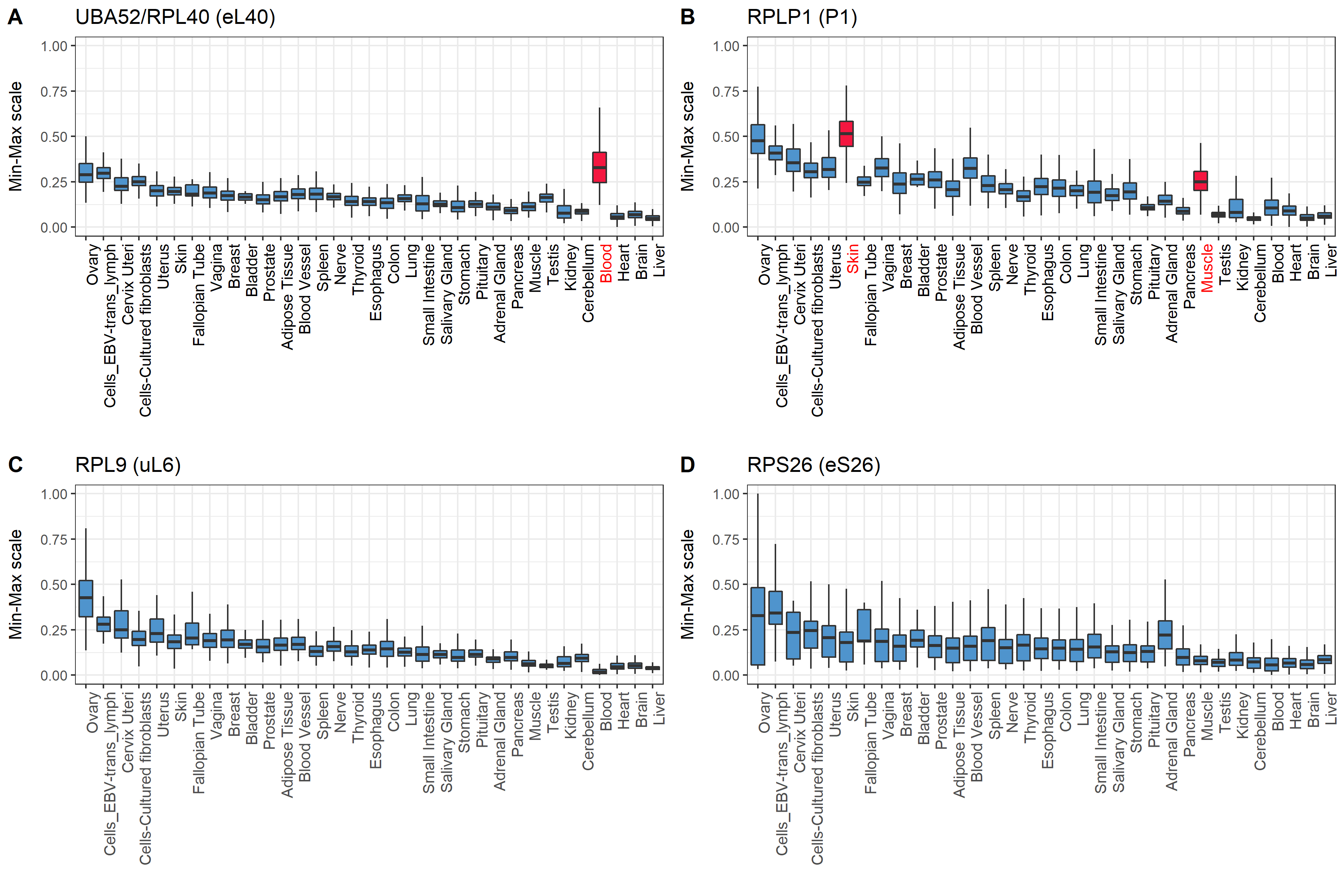


**Supplementary Figure S12.** Boxplots of tissue-enriched (**A**) UBA52/RPL40-precursor/eL40 (enriched in blood) and (**B)** RPLP1/P1 (enriched in muscle and skin), as well as constitutively expressed (**C**) RPL9/uL6 and (**D**) RPS26/eS26, presenting their expression profiles across 33 human tissue categories of GTEx project (19). Expression values were scaled between 0 and 1 using Min-Max normalization. Tissue names, and corresponding boxplots, with enriched RP expression are highlighted in red.


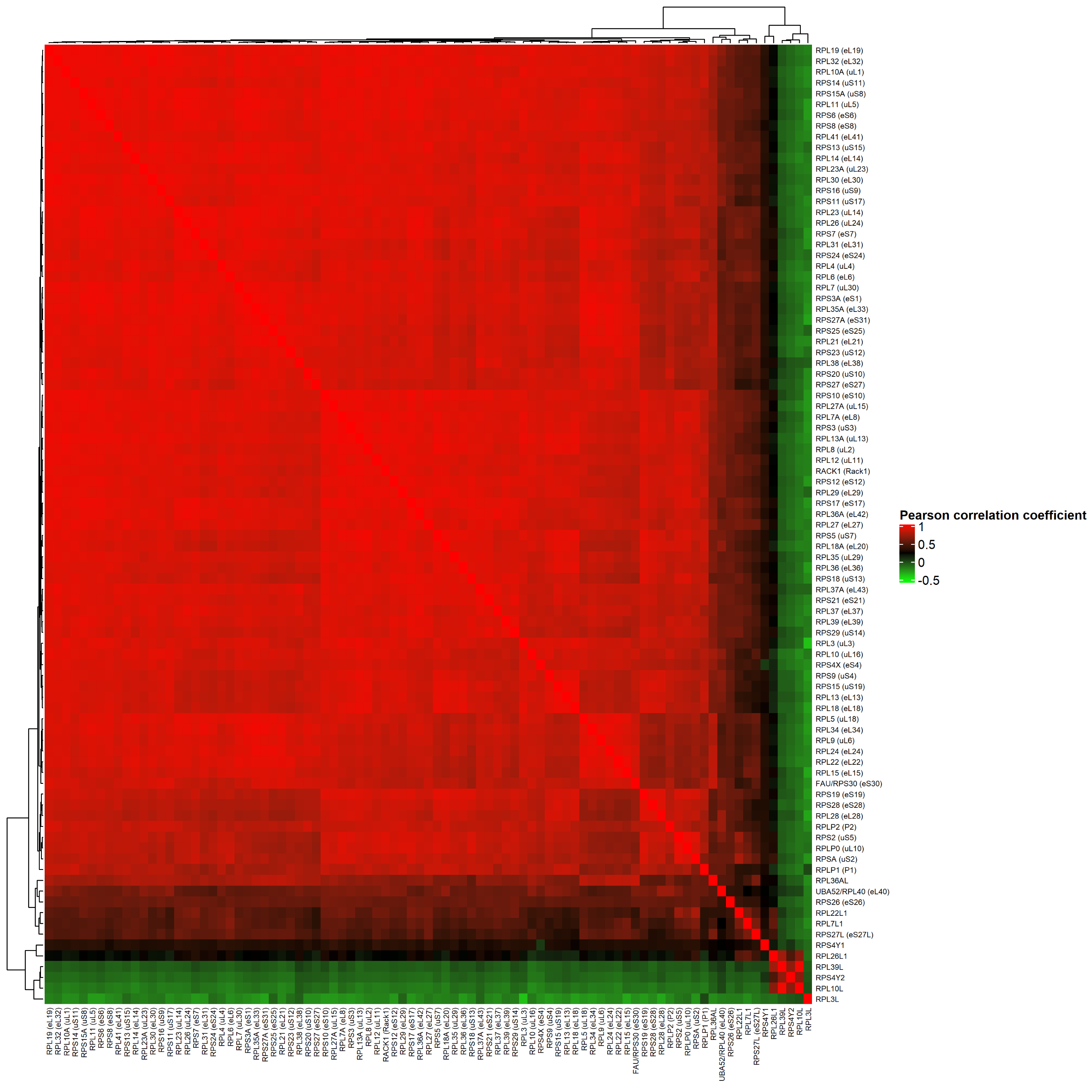


**Supplementary Figure S13.** Correlation (Pearson correlation coefficients) of 89 RP expression profiles (TPM) for human tissues of GTEx project (19). RPs (x- and y-axis) are ordered based on hierarchical clustering (complete agglomeration method). Heatmap was created using ComplexHeatmap (20).


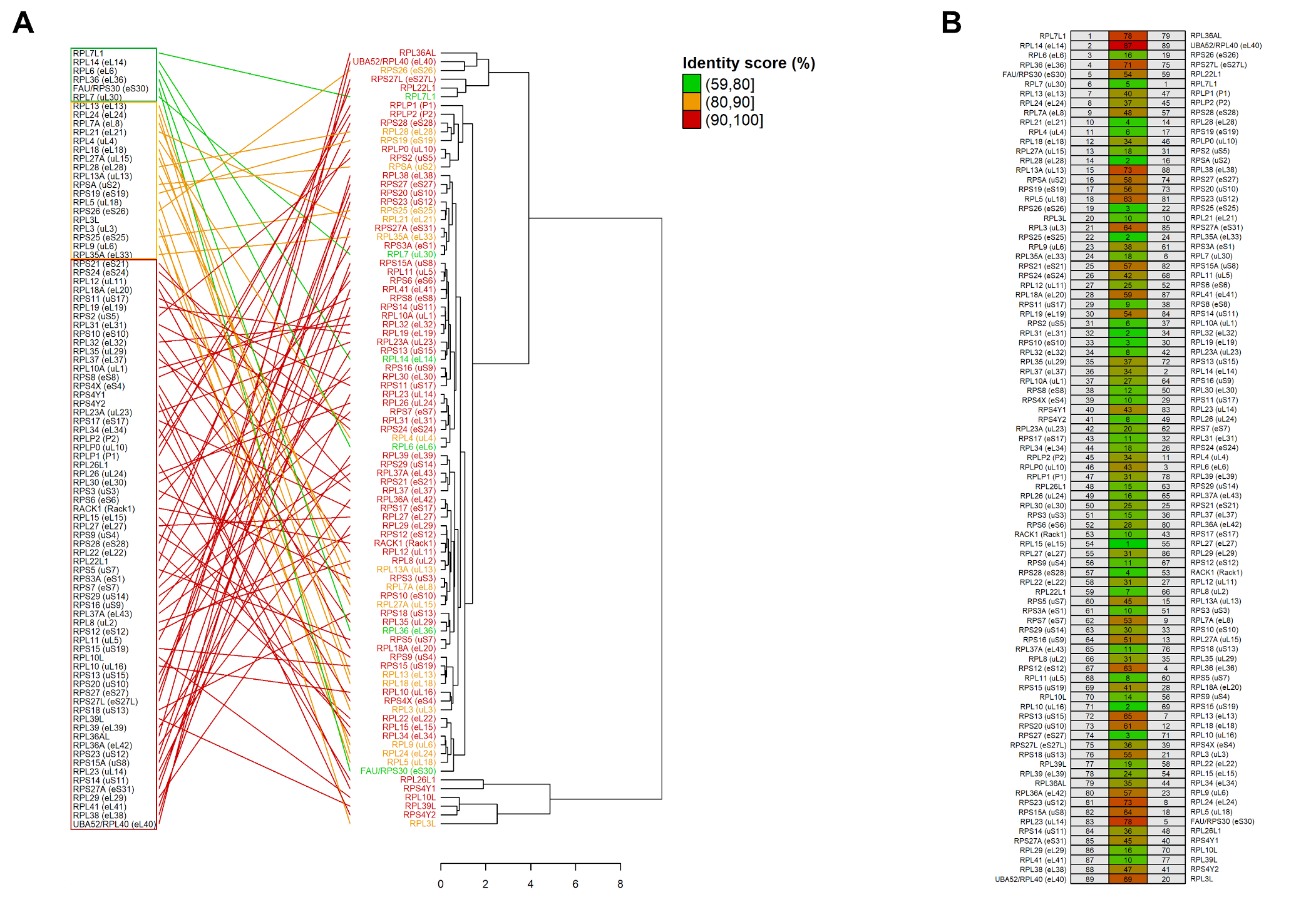


**Supplementary Figure 14.** Relationship between sequence conservation and human tissue expression profiles, from GTEx project (19), for 89 RPs. (A) Tanglegram of increasing, within-cluster RP identity scores (left) and hierarchical clustering (complete agglomeration method) of RP Pearson correlation coefficient matrix (correlation patterns) (right). RPs and their connections are colored based on a within-cluster identity score scale with green for 59-80%, orange for 80-90% and red for 90-100%. RPs were repeatedly split to an increasing number of clusters (from 2 up to 20), based on their correlation patterns, and identity scores between clusters were compared using Kruskal-Wallis rank sum test, exhibiting lack of statistically significant differences (p-value>0.05) (not shown). Tanglegram was created using dendextend (22). (B) Ranking of RPs based on within-cluster identity score (left) and correlation patterns (right). Absolute values of rank differences are shown in the middle with a gradient color scale (green-red). Test for Spearman correlation between paired samples also exhibited lack of significant correlation between the pairwise rank lists (Spearman's rank correlation rho: -0.16; p-value>0.05).

References

1. Lecompte,O., Ripp,R., Thierry,J.C., Moras,D. and Poch,O. (2002) Comparative analysis of ribosomal proteins in complete genomes: An example of reductive evolution at the domain scale. *Nucleic Acids Res.*, **30**, 5382–5390.

2. Melnikov,S., Manakongtreecheep,K. and Söll,D. (2018) Revising the structural diversity of ribosomal proteins across the three domains of life. *Mol. Biol. Evol.*, **35**, 1588–1598.

3. Anger,A.M., Armache,J.P., Berninghausen,O., Habeck,M., Subklewe,M., Wilson,D.N. and Beckmann,R. (2013) Structures of the human and Drosophila 80S ribosome. *Nature*, **497**, 80–85.

4. Kirn-Safran,C.B., Oristian,D.S., Focht,R.J., Parker,S.G., Vivian,J.L. and Carson,D.D. (2007) Global growth deficiencies in mice lacking the ribosomal protein HIP/RPL29. *Dev. Dyn.*, **236**, 447–460.

5. Hoke,D.E., LaBrenz,S.R., Hook,M. and Carson,D.D. (2000) Multiple domains contribute to heparin/heparan sulfate binding by human HIP/L29. *Biochemistry*, **39**, 15686–15694.

6. Liu,S., Zhou,F., Hook,M. and Carson,D.D. (1997) A heparin-binding synthetic peptide of heparin/heparan sulfate-interacting protein modulates blood coagulation activities. *Proc. Natl. Acad. Sci. U. S. A.*, **94**, 1739–1744.

7. Hoke,D.E., Regisford,E.G., Julian,J., Amin,A., Begue-Kirn,C. and Carson,D.D. (1998) Murine HIP/L29 is a heparin-binding protein with a restricted pattern of expression in adult tissues. *J. Biol. Chem.*, **273**, 25148–25157.

8. Espinar-Marchena,F., Rodríguez-Galán,O., Fernández-Fernández,J., Linnemann,J. and de la Cruz,J. (2018) Ribosomal protein L14 contributes to the early assembly of 60S ribosomal subunits in Saccharomyces cerevisiae. *Nucleic Acids Res.*, **46**, 4715–4732.

9. Stelter,P., Huber,F.M., Kunze,R., Flemming,D., Hoelz,A. and Hurt,E. (2015) Coordinated Ribosomal L4 Protein Assembly into the Pre-Ribosome Is Regulated by Its Eukaryote-Specific Extension. *Mol. Cell*, **58**, 854–862.

10. Shen,C.-L., Liu,C.-D., You,R.-I., Ching,Y.-H., Liang,J., Ke,L., Chen,Y.-L., Chen,H.-C., Hsu,H.-J., Liou,J.-W., *et al.* (2016) Ribosome Protein L4 is essential for Epstein-Barr Virus Nuclear Antigen 1 function. *Proc. Natl. Acad. Sci. U. S. A.*, **113**, 2229–2234.

11. Edgar,R.C. (2004) MUSCLE: multiple sequence alignment with high accuracy and high throughput. *Nucleic Acids Res.*, **32**, 1792–1797.

12. Bodenhofer,U., Bonatesta,E., Horejš-Kainrath,C. and Hochreiter,S. (2015) msa: an R package for multiple sequence alignment. *Bioinformatics*, **31**, 3997–3999.

13. Thibaud-Nissen,F., Souvorov,A., Murphy,T., DiCuccio,M. and Kitts,P. (2013) Eukaryotic Genome Annotation Pipeline. In *The NCBI Handbook. 2nd edition.* Bethesda (MD): National Center for Biotechnology Information (US), pp. 111–130.

14. Papadopoulos,J.S. and Agarwala,R. (2007) COBALT: constraint-based alignment tool for multiple protein sequences. *Bioinformatics*, **23**, 1073–1079.

15. Waterhouse,A.M., Procter,J.B., Martin,D.M.A., Clamp,M. and Barton,G.J. (2009) Jalview Version 2—a multiple sequence alignment editor and analysis workbench. *Bioinformatics*, **25**, 1189–1191.

16. Lu,S., Wang,J., Chitsaz,F., Derbyshire,M.K., Geer,R.C., Gonzales,N.R., Gwadz,M., Hurwitz,D.I., Marchler,G.H., Song,J.S., *et al.* (2020) CDD/SPARCLE: the conserved domain database in 2020. *Nucleic Acids Res.*, **48**, D265–D268.

17. Yates,A.D., Achuthan,P., Akanni,W., Allen,J., Allen,J., Alvarez-Jarreta,J., Amode,M.R., Armean,I.M., Azov,A.G., Bennett,R., *et al.* (2019) Ensembl 2020. *Nucleic Acids Res.*, **48**, D682–D688.

18. Barbosa-Morais,N.L., Irimia,M., Pan,Q., Xiong,H.Y., Gueroussov,S., Lee,L.J., Slobodeniuc,V., Kutter,C., Watt,S., Colak,R., *et al.* (2012) The evolutionary landscape of alternative splicing in vertebrate species. *Science*, **338**, 1587–93.

19. Lonsdale,J., Thomas,J., Salvatore,M., Phillips,R., Lo,E., Shad,S., Hasz,R., Walters,G., Garcia,F., Young,N., *et al.* (2013) The Genotype-Tissue Expression (GTEx) project. *Nat. Genet.*, **45**, 580–585.

20. Gu,Z., Eils,R. and Schlesner,M. (2016) Complex heatmaps reveal patterns and correlations in multidimensional genomic data. *Bioinformatics*, **32**, 2847–2849.

21. Everaert,C., Volders,P.-J., Morlion,A., Thas,O. and Mestdagh,P. (2020) SPECS: a non-parametric method to identify tissue-specific molecular features for unbalanced sample groups. *BMC Bioinformatics*, **21**, 58.

22. Galili,T. (2015) dendextend: an R package for visualizing, adjusting and comparing trees of hierarchical clustering. *Bioinformatics*, **31**, 3718–3720.
